# A second update on mapping the human genetic architecture of COVID-19

**DOI:** 10.1038/s41586-023-06355-3

**Published:** 2023-09-06

**Authors:** The COVID-19 Host Genetics Initiative, Masahiro Kanai, Shea J. Andrews, Mattia Cordioli, Christine Stevens, Benjamin M. Neale, Mark Daly, Andrea Ganna, Karolina Chwialkowska, Amy Trankiem, Mary K. Balaconis, Matthew Solomonson

**Affiliations:** 1 Broad Institute of MIT and Harvard, Cambridge, MA USA; 2 University of California San Francisco, San Francisco, CA USA; 3 Institute for Molecular Medicine Finland (FIMM), University of Helsinki, Helsinki, Finland; 4 Analytic and Translational Genetics Unit, Massachusetts General Hospital, Boston, MA USA; 5 Department of Psychiatry, School of Medicine, Yale University, New Haven, CT USA; 6 Veteran Affairs Connecticut Healthcare System, West Haven, CT USA; 7 Yale University, New Haven, CT USA; 8 Centre for Bioinformatics and Data Analysis, Medical University of Bialystok, Bialystok, Poland; 9 Department of Computational Medicine and Bioinformatics, University of Michigan, Ann Arbor, MI USA; 10 Faculty of Industrial Sciences and Technology, Universiti Malaysia Pahang, Pahang, Malaysia; 11 Icahn School of Medicine at Mount Sinai, New York, NY USA; 12 Genolier Innovation Network and Hub, Swiss Medical Network, Genolier Healthcare Campus, Genolier, Switzerland; 13 Immediate, Independent Multidisciplinary Med & Biotech Research Association, Milan, Italy; 14 Clinical Research Unit of Nanoro, Institut de Recherche en Sciences de la Santé, Nanoro, Burkina Faso; 15 Sydney Brenner Institute for Molecular Biosciences, University of the Witwatersrand, Johannesburg, South Africa; 16 McGill University, Montreal, Quebec Canada; 17 University of Peurto Rico, San Juan, PR USA; 18 Institute for Biomedical Technologies, National Research Council, Milan, Italy; 19 University of Miami, Miami, FL USA; 20 Department of Biochemistry, All India Institute of Medical Sciences, Kalyani, India; 21 Department of Biosciences, School of Science and Technology, Nottingham Trent University, Nottingham, UK; 22 Department of Clinical Pathology, Faculty of Medicine, Mansoura University, Mansoura, Egypt; 23 Department of Clinical Pathology, Faculty of Medicine, Tanta University, Tanta, Egypt; 24 Neuron23, San Francisco, CA USA; 25 Vanderbilt University, Nasvhille, TN USA; 26 Stroke Pharmacogenomics and Genetics, Institut d’Investigació Biomèdica Sant Pau (IIBSANT PAU), Barcelona, Spain; 27 Department of Medicine, Surgery and Health Sciences, University of Trieste, Trieste, Italy; 28 Program in Medical and Population Genetics, Broad Institute of MIT and Harvard, Cambridge, MA USA; 29 Department of Genetics, University Medical Centre Groningen, University of Groningen, Groningen, The Netherlands; 30 Departamento de Tecnología Médica, Facultad de Ciencias de la Salud, Universidad de Tarapacá, Arica, Chile; 31 King’s College London, London, UK; 32 Naina Tech, New York, NY USA; 33 Vrije Universiteit Amsterdam, Amsterdam, The Netherlands; 34 Medical School, University of Pécs, Pécs, Hungary; 35 MNM Diagnostics, Poznan, Poland; 36 Genomics England, London, UK; 37 Heart Institute (InCor), University of Sao Paulo Medical School, Sao Paulo, Brazil; 38 Department of Physiology, All India Institute of Medical Sciences, Kalyani, India; 39 Montreal Heart Institute, Montreal, Quebec Canada; 40 Nantes Université, Ecole Centrale Nantes, INSERM, Center for Research in Transplantation and Translational Immunology, Nantes, France; 41 Department of Computer Science and Technology, University of Cambridge, Cambridge, UK; 42 William Harvey Research Institute, Barts and the London School of Medicine and Dentistry, Queen Mary University of London, London, UK; 43 University of Helsinki, Helsinki, Finland; 44 Department of Internal Medicine, Erasmus MC, University Medical Center Rotterdam, Rotterdam, The Netherlands; 45 Fonds de la Recherche Scientifique (FNRS) & Centre de Génétique Humaine, Hôpital Erasme, Université Libre de Bruxelles, Brussels, Belgium; 46 Department of Genetics, Hôpital Erasme, Université Libre de Bruxelles, Brussels, Belgium; 47 All India Institute of Medical Sciences Kalyani, Kalyani, India; 48 National Laboratory of Genomics for Biodiversity, CINVESTAV, Guanajuato, Mexico; 49 Queensland University of Technology, Brisbane, Queensland Australia; 50 Department of Human Genetics, McGill University, Montreal, Quebec Canada; 51 Lady Davis Institute, Jewish General Hospital, McGill University, Montreal, Quebec Canada; 52 Kyoto-McGill International Collaborative School in Genomic Medicine, Graduate School of Medicine, Kyoto University, Kyoto, Japan; 53 Japan Society for the Promotion of Science, Tokyo, Japan; 54 Division of Life Sciences, University of Northampton, Northampton, UK; 55 Root Deep, Boston, MA USA; 56 Instituto do Coração, Sao Paulo, Brazil; 57 Stanford University, Stanford, CA USA; 58 Department of Internal Medicine, Erasmus MC, University Medical Center Rotterdam, Rotterdam, The Netherlands; 59 Department of Epidemiology, Erasmus MC, University Medical Center Rotterdam, Rotterdam, The Netherlands; 60 Institute for Community Medicine, University Medicine Greifswald, Greifswald, Germany; 61 GI and Liver Section, Virgen del Rocío University Hospital and Department of Medicine, University of Seville, Institute of Biomedicine of Seville, Seville, Spain; 62 CIBEREHD, Instituto de Salud Carlos III (ISCIII), Seville, Spain; 63 Department of Health, Traunstein, Germany; 64 Rutgers Cancer Institute of New Jersey, New Brunswick, NJ USA; 65 Erasmus Medical Center, Rotterdam, The Netherlands; 66 Institute of Psychiatric Phenomics & Genomics, University of Munich, Munich, Germany; 67 Department of Psychiatry, University of Munich, Munich, Germany; 68 Institute of Virology, Technical University of Munich/Helmholtz Munich, Munich, Germany; 69 Institute of Human Genetics, University Hospital, Faculty of Medicine, University of Bonn, Bonn, Germany; 70 Department of Psychiatry, University Hospital, Faculty of Medicine, University of Bonn, Bonn, Germany; 71 Digital Health Center, Hasso Plattner Institute, University of Potsdam, Potsdam, Germany; 72 Institute of Biomedicine and Cancer Research Laboratories, Western Cancer Centre FICAN West, University of Turku, Turku, Finland; 73 IRCCS Istituto Giannina Gaslini, Genova, Italy; 74 Department of Neurosciences, Rehabilitation, Ophthalmology, Genetics, Maternal and Child Health, University of Genova, Genova, Italy; 75 Department of Biomedical Data Science, Stanford University, Stanford, CA USA; 76 Fundación Pública Andaluza para la Gestión de la Investigación en Salud de Sevilla, Seville, Spain; 77 Department of Pediatrics, Stanford University, Stanford, CA USA; 78 Department of Internal Medicine, Erasmus MC, University Medical Center Rotterdam, Rotterdam, The Netherlands; 79 University Medical Center Utrecht, Utrecht, The Netherlands; 80 Instituto de Investigación Interdisciplinaria & Escuela de Medicina, Universidad de Talca, Talca, Chile; 81 Programa de Genética Humana, ICBM and Chile Departamento de Oncología Básico Clínica, Universidad de Chile, Talca, Chile; 82 Department of Statistical Genetics, Osaka University Graduate School of Medicine, Osaka, Japan; 83 Department of Internal Medicine, International Islamic University Malaysia, Pahang, Malaysia; 84 Centro para el Desarrollo de la Investigación Científica (CEDIC), Asunción, Paraguay; 85 23andMe, Sunnyvale, CA USA; 86 Washington University School Of Medicine, St Louis, MO USA; 87 Department of Psychological & Brain Sciences, Washingon University in St Louis, St Louis, MO USA; 88 Institute of Biological Psychiatry, Mental Health Centre Sct Hans, Mental Health Services Capital Region of Denmark, Copenhagen, Denmark; 89 iPSYCH, The Lundbeck Foundation Initiative for Integrative Psychiatric Research, Aarhus, Denmark; 90 Laureate Institute for Brain Research, Tulsa, OK USA; 91 Department of Radiology, UCSD, San Diego, CA USA; 92 Nork Infection Clinical Hospital, Yerevan, Armenia; 93 Institute of Molecular Biology NAS RA, Yerevan, Armenia; 94 National Research Center for Cardiac Surgery, Astana, Kazakhstan; 95 City Infectious Disease Center, Astana, Kazakhstan; 96 National Center for Biotechnology, Astana, Kazakhstan; 97 Health Department of Astana, Astana, Kazakhstan; 98 MRC Integrative Epidemiology Unit, University of Bristol, Bristol, UK; 99 Division of Psychitary, University of Edinburgh, Edinburgh, UK; 100 Institute for Molecular Medicine Finland (FIMM), Helsinki, Finland; 101 University of Liege, GIGA-Institute, Liege, Belgium; 102 CHC Mont-Légia, Liege, Belgium; 103 BHUL (Liège Biobank), CHU of Liège, Liege, Belgium; 104 Institute for Molecular Medicine Finland, University of Helsinki, Helsinki, Finland; 105 Analytic & Translational Genetics Unit, Massachusetts General Hospital, Boston, MA USA; 106 Stanley Center for Psychiatric Research, Broad Institute of MIT and Harvard, Cambridge, MA USA; 107 CHU of Liege, Liege, Belgium; 108 University of Liege, Liege, Belgium; 109 Centre de Génétique Humaine, Hôpital Erasme, Université Libre de Bruxelles, Brussels, Belgium; 110 Service de Médecine Interne, Hôpital Erasme, Université Libre de Bruxelles, Brussels, Belgium; 111 McGill Genome Centre and Department of Human Genetics, McGill University, Montréal, Quebec Canada; 112 Department of Human Genetics, Epidemiology, Biostatistics and Occupational Health, McGill University, Montreal, Quebec Canada; 113 Department of Twin Research, King’s College London, London, UK; 114 The Meakins-Christie Laboratories at the Research Institute of the McGill University Heath Centre Research Institute and Department of Medicine, Faculty of Medicine, McGill University, Montreal, Quebec Canada; 115 Research Centre of the Centre Hospitalier de l’Université de Montréal (CRCHUM), Montreal, Quebec Canada; 116 Centre Hospitalier de l’Université de Montréal (CHUM), Montreal, Quebec Canada; 117 Department of Epidemiology, Biostatistics and Occupational Health, McGill University, Montreal, Quebec Canada; 118 Department of Emergency Medicine, McGill University, Montreal, Quebec Canada; 119 Emergency Department, Jewish General Hospital, McGill University, Montreal, Quebec Canada; 120 McGill AIDS Centre, Department of Microbiology and Immunology, Lady Davis Institute for Medical Research, Jewish General Hospital, McGill University, Montreal, Quebec Canada; 121 Division of Infectious Diseases, Lausanne University Hospital and University of Lausanne, Lausanne, Switzerland; 122 Research Centre of the Centre Hospitalier de l’Université de Montréal and Université de Montréal, Montreal, Quebec Canada; 123 School of Rehabilitation, Faculty of Medicine and Health Sciences, Université de Sherbrooke, Sherbrooke, Canada; 124 Department of Medicine, Faculty of Medicine, Université de Montréal, Montreal, Quebec Canada; 125 Center for Immunity, Inflammation and Infectious Diseases, Montreal Clinical Research Institute (IRCM), Montreal, Quebec Canada; 126 Génome Québec, Montreal, Quebec Canada; 127 Département de Microbiologie et Infectiologie, Université de Sherbrooke, Sherbrooke, Quebec Canada; 128 Département de Médecine, Service d’Infectiologie, Centre de Recherche Clinique du Centre Hospitalier Universitaire de Sherbrooke, Sherbrooke, Quebec Canada; 129 Department of Epidemiology, Biostatistics and Occupational Health, McGill University, Montreal, Quebec Canada; 130 Center for Clinical Epidemiology, Lady Davis Institute, Jewish General Hospital, Montreal, Quebec Canada; 131 Department of Neurology and Neurosurgery, McGill University, Montreal, Quebec Canada; 132 Centre Intégré Universitaire de Santé et de Services Sociaux (CIUSSS) du Saguenay–Lac-Saint-Jean, Saguenay, Quebec Canada; 133 Department of Pharmacology-Physiology, Medicine and Health Sciences Faculty, Université de Sherbrooke, Sherbrooke, Quebec Canada; 134 Division of Respiratory Medicine, Department of Pediatrics, Sainte-Justine University Hospital Center, University of Montreal, Montreal, Quebec Canada; 135 Centre of Genomics and Policy, McGill University, Montreal, Quebec Canada; 136 Division of Genetic Medicine, Department of Medicine, Vanderbilt University Medical Center, Nashville, TN USA; 137 Vanderbilt Genetics Institute, Vanderbilt University Medical Center, Nashville, TN USA; 138 Institute of Human Genetics, University Hospital Bonn, Medical Faculty University of Bonn, Bonn, Germany; 139 Institute of Genomic Statistics and Bioinformatics, University Hospital Bonn, Medical Faculty University of Bonn, Bonn, Germany; 140 Department of Gastroenterology, Hepatology and Infectious Diseases, University Hospital Duesseldorf, Medical Faculty Heinrich Heine University, Duesseldorf, Germany; 141 Department of Gastroenterology, Hepatology and Infectiology, University Hospital Magdeburg, Magdeburg, Germany; 142 Clinic for Cardiology, Angiology and Internal Intensive Medicine, Medical Clinic I, RWTH Aachen University, Aachen, Germany; 143 Department of Pneumology and Intensive Care Medicine, Faculty of Medicine, RWTH Aachen University, Aachen, Germany; 144 Department of Pneumology, Hannover Medical School, Hannover, Germany; 145 Department of Gastroenterology, Hepatology and Endocrinology, Hannover Medical School, Hannover, Germany; 146 Hannover Unified Biobank, Hannover Medical School, Hannover, Germany; 147 Department I of Internal Medicine, Faculty of Medicine and University Hospital of Cologne, University of Cologne, Cologne, Germany; 148 Center for Molecular Medicine Cologne (CMMC), University of Cologne, Cologne, Germany; 149 German Center for Infection Research (DZIF), partner site Bonn-Cologne, Cologne, Germany; 150 Institute for Human Genetics and Genomic Medicine, Medical Faculty, RWTH Aachen University, Aachen, Germany; 151 Department of Anesthesiology and Intensive Care Medicine, University Hospital Essen, University Duisburg-Essen, Essen, Germany; 152 Department of Child and Adolescent Psychiatry, University Hospital Essen, University of Duisburg-Essen, Essen, Germany; 153 Department of Infectious Diseases, University Hospital Essen, University Duisburg-Essen, Essen, Germany; 154 Department of Pneumology, Allergology and Respiratory Medicine, University Hospital Saarland, Homburg, Germany; 155 Center of Human and Molecular Biology, Department of Human Genetics, University Hospital Saarland, Homburg, Germany; 156 Department of Genetics & Epigenetics, Saarland University, Saarbrücken, Germany; 157 Institute of Innate Immunity, University Hospital Bonn, Bonn, Germany; 158 University of Toronto, Toronto, Ontario Canada; 159 Dalla Lana School of Public Health, University of Toronto, Toronto, Ontario Canada; 160 Lunenfeld-Tanenbaum Research Institute, Sinai Health, Toronto, Ontario Canada; 161 Simon Fraser University, Burnaby, British Columbia Canada; 162 Lady Davis Institute, Jewish General Hospital, McGill University, Montreal, Quebec Canada; 163 University of Waterloo, Waterloo, Ontario Canada; 164 The Hospital for Sick Children, Toronto, Ontario Canada; 165 Queen’s University, Kingston, Ontario Canada; 166 Mount Sinai Hospital, Sinai Health, Toronto, Ontario Canada; 167 St Michael’s Hospital, Unity Health Toronto, Toronto, Ontario Canada; 168 Princess Margaret Cancer Center, University Health Network, Toronto, Ontario Canada; 169 Ontario Institute for Cancer Research, Toronto, Ontario Canada; 170 Dynacare Laboratory, Toronto, Ontario Canada; 171 William Osler Health System, Brampton, Ontario Canada; 172 University Health Network, Toronto, Ontario Canada; 173 Mackenzie Health, Toronto, Ontario Canada; 174 Women’s College Hospital, Toronto, Ontario Canada; 175 University of British Columbia, Vancouver, British Columbia Canada; 176 BC Children’s Hospital, Vancouver, British Columbia Canada; 177 St Paul’s Hospital, Vancouver, British Columbia Canada; 178 University of Calgary, Calgary, Alberta Canada; 179 Alberta Children’s Hospital, Calgary, Alberta Canada; 180 British Columbia Cancer Research Centre, Vancouver, British Columbia Canada; 181 Toronto General Hospital, Toronto, Ontario Canada; 182 Division of Infectious Diseases, Department of Medicine, University of Ottawa, Ottawa, Ontario Canada; 183 Department of Biochemistry, Microbiology and Immunology, University of Ottawa Centre of Infection, Immunity and Inflammation (CI3), Ottawa, Ontario Canada; 184 The Ottawa Hospital Research Institute, Ottawa, Ontario Canada; 185 University of Montreal, Montreat, Quebec Canada; 186 Ste-Justine University Health Centre, Montreal, Quebec Canada; 187 University of Toronto Ontario, Toronto, Ontario Canada; 188 MitogenDx/Eve Technologies, Toronto, Ontario Canada; 189 Genome Sciences Centre, BC Cancer, Vancouver, British Columbia Canada; 190 Montreal Children’s Hospital, Montreal, Quebec Canada; 191 London Health Sciences Centre, London, Ontario Canada; 192 Kidney and Metabolism Program, St Michael’s Hospital, Unity Health Toronto, Toronto, Ontario Canada; 193 Sinai Health, Mount Sinai Hospital, Toronto, Ontario Canada; 194 Baycrest Health Sciences, Toronto, Ontario Canada; 195 Anschutz Medical Campus, University of Colorado, Aurora, CO USA; 196 Institute of Psychiatric Phenomics and Genomics (IPPG), University Hospital, LMU Munich, Munich, Germany; 197 Department of Genetic Epidemiology in Psychiatry, Central Institute of Mental Health, Medical Faculty Mannheim, University of Heidelberg, Mannheim, Germany; 198 Department of Psychiatry and Behavioral Sciences, Johns Hopkins University, Baltimore, MD USA; 199 Department of Psychiatry and Behavioral Sciences, SUNY Upstate Medical University, Syracuse, NY USA; 200 Department of Psychiatry and Psychotherapy, University Hospital, LMU Munich, Munich, Germany; 201 Institute of Virology, Technical University Munich/Helmholtz Zentrum München, Munich, Germany; 202 Department of Psychiatry and Psychotherapy, University Hospital Bonn, Medical Faculty University of Bonn, Bonn, Germany; 203 CIBER de Salud Mental, Instituto de Salud Carlos III, Barcelona, Spain; 204 Munich Clinic Schwabing, Academic Teaching Hospital, Ludwig-Maximilians University of Munich (LMU), Munich, Germany; 205 Department of Internal Medicine II, University Hospital rechts der Isar, Technical University of Munich, School of Medicine, Munich, Germany; 206 Institute of Clinical Chemistry and Pathobiochemistry, School of Medicine, Technical University of Munich, Munich, Germany; 207 TranslaTUM, Center for Translational Cancer Research, Technical University of Munich, Munich, Germany; 208 Department of Psychiatry and Psychotherapy, University Medical Center Goettingen, Goettingen, Germany; 209 German Center for Neurodegenerative Diseases (DZNE), Goettingen, Germany; 210 Neurosciences and Signaling Group, Institute of Biomedicine (iBiMED), Department of Medical Sciences, University of Aveiro, Agra do Crasto, Aveiro, Portugal; 211 Munich Site of the German Center for Infection Research (DZIF), Munich, Germany; 212 Institute of Computational Biology, Helmholtz Center Munich, Oberschleissheim, Germany; 213 Department of Hygiene and Epidemiology, University of Ioannina School of Medicine, Ioannina, Greece; 214 1st Department of Internal Medicine and Infectious Diseases Unit, University Hospital of Ioannina, Ioannina, Greece; 215 Department of Epidemiology and Biostatistics, School of Public Health, Imperial College London, London, UK; 216 Center for Evidence-Based Medicine, Department of Health Services, Policy and Practice, School of Public Health, Brown University, Providence, RI USA; 217 University of Ioannina School of Medicine, Ioannina, Greece; 218 Centre for Systems Biology, Biomedical Research Foundation of the Academy of Athens, Athens, Greece; 219 Institute of Clinical Molecular Biology, Christian-Albrechts-University, Kiel, Germany; 220 Novo Nordisk Foundation Center for Protein Research, Faculty of Health and Medical Sciences, University of Copenhagen, Copenhagen, Denmark; 221 Institut de Biotecnologia i de Biomedicina, Universitat Autònoma de Barcelona, Barcelona, Spain; 222 ICREA, Barcelona, Spain; 223 Institute of Biotechnology, Life Science Centre, Vilnius University, Vilnius, Lithuania; 224 Research Group for Evolutionary Immunogenomics, Max Planck Institute for Evolutionary Biology, Plön, Germany; 225 Research Unit for Evolutionary Immunogenomics, Department of Biology, University of Hamburg, Hamburg, Germany; 226 Department of Gastroenterology, Hospital Universitario Ramón y Cajal, University of Alcalá, Instituto Ramón y Cajal de Investigación Sanitaria (IRYCIS), Madrid, Spain; 227 Centro de Investigación Biomédica en Red en Enfermedades Hepáticas y Digestivas (CIBEREHD), Instituto de Salud Carlos III (ISCIII), Madrid, Spain; 228 Vall d’Hebron Institut de Recerca (VHIR), Vall d’Hebron Hospital Universitari, Barcelona, Spain; 229 Fondazione IRCCS Ca’ Granda Ospedale Maggiore Policlinico, Milan, Italy; 230 Department of Medicine, Møre & Romsdal Hospital Trust, Ålesund, Norway Geminicenter for Sepsis Research, Institute of Circulation and Medical Imaging (ISB), NTNU, Trondheim, Norway; 231 Microbiology Unit, Hospital Universitario Clinico San Cecilio, Granada, Spain; 232 Ibs.Granada Instituto de Investigación Biosanitaria, Granada, Spain; 233 Klinik für Innere Medizin I, Universitätsklinikum Schleswig-Holstein, Campus Kiel, Kiel, Germany; 234 Emergency Department, University Hospital Regensburg, Regensburg, Germany; 235 Department for Infectious Diseases and Infection Control, University Hospital Regensburg, Regensburg, Germany; 236 Department of Medicine I, Gastroenterology, Hepatology and Endocrinology, Medical University of Innsbruck, Innsbruck, Austria; 237 Christian Doppler Laboratory of Iron and Phosphate Biology at the Department of Medicine I, Medical University of Innsbruck, Innsbruck, Austria; 238 Institute of Clinical Medicine, University of Oslo, Oslo, Norway; 239 Department of Microbiology, Oslo University Hospital, Oslo, Norway; 240 Hospital Clinic, University of Barcelona and IDIBAPS, Barcelona, Spain; 241 European Foundation for the Study of Chronic Liver Failure (EF-CLIF), Barcelona, Spain; 242 GCAT—Genomes for Life, Germans Trias i Pujol Health Sciences Research Institute (IGTP), Badalona, Spain; 243 Genomes for Life—GCAT Laboratory, Germans Trias i Pujol Research Institute (IGTP), Spainermans Trias i Pujol Research Institute (IGTP), Badalona, Spain; 244 University of Milan, Milan, Italy; 245 Department of Liver and Gastrointestinal Diseases, Biodonostia Health Research Institute, Donostia University Hospital, University of the Basque Country (UPV/EHU), CIBEREHD, Ikerbasque, San Sebastian, Spain; 246 Digestive Diseases Unit, Virgen del Rocio University Hospital, Institute of Biomedicine of Seville, University of Seville, Seville, Spain; 247 University of Sevilla, Seville, Spain; 248 Instituto de Biomedicina de Sevilla (IBIS), Seville, Spain; 249 Hospital Universitario Virgen del Rocío de Seville, Seville, Spain; 250 Universitat Autònoma de Barcelona, Bellatera, Spain; 251 Liver Unit, Department of Internal Medicine, Hospital Universitari Vall d’Hebron, Vall d’Hebron Barcelona Hospital Campus, Barcelona, Spain; 252 Gastrointestinal Genetics Lab, CIC bioGUNE—BRTA, Derio, Spain; 253 Ikerbasque, Basque Foundation for Science, Bilbao, Spain; 254 Department of Liver and Gastrointestinal Diseases, Biodonostia Health Research Institute—Donostia University Hospital, University of the Basque Country (UPV/EHU), CIBEREHD, Ikerbasque, San Sebastian, Spain Ikerbasque, Basque Foundation for Science, Bilbao, Spain; 255 Department I of Internal Medicine, Faculty of Medicine and University Hospital Cologne, University of Cologne, Cologne, Germany; 256 University of Cologne, Faculty of Medicine and University Hospital Cologne, Cologne Excellence Cluster on Cellular Stress Responses in Aging-Associated Diseases (CECAD), Cologne, Germany; 257 European Reference Network on Hepatological Diseases (ERN RARE-LIVER), Fondazione IRCCS San Gerardo dei Tintori, Monza, Italy; 258 Division of Gastroenterology, Center for Autoimmune Liver Diseases, School of Medicine and Surgery, University of Milano-Bicocca, Milan, Italy; 259 Genomes for Life—GCAT lab, Germans Trias i Pujol Research Institute (IGTP), Badalona, Spain; 260 IRCCS Humanitas Research Hospital, Milan, Italy; 261 Department of Biomedical Sciences, Humanitas University, Pieve Emanuele, Milan, Italy; 262 Zentrum für Humangenetik Regensburg, Regensburg, Germany; 263 University Hospital Schleswig-Holstein (UKSH), Campus Kiel, Kiel, Germany; 264 Section for Gastroenterology, Department of Transplantation Medicine, Division for Cancer Medicine, Surgery and Transplantation, Oslo University Hospital Rikshospitalet, Oslo, Norway; 265 Research Institute for Internal Medicine, Division of Surgery, Inflammatory Diseases and Transplantation, Oslo University Hospital Rikshospitalet and University of Oslo, Oslo, Norway; 266 Norwegian PSC Research Center, Department of Transplantation Medicine, Division of Surgery, Inflammatory Diseases and Transplantation, Oslo University Hospital Rikshospitalet, Oslo, Norway; 267 Private University in the Principality of Liechtenstein, Triesen, Liechtenstein; 268 Division of Rheumatology, Inflammation and Immunity, Brigham and Women’s Hospital and Harvard Medical School, Boston, MA USA; 269 Division of Genetics, Department of Medicine, Brigham and Women’s Hospital, Boston, MA USA; 270 Department of Biomedical Informatics, Harvard Medical School, Boston, MA USA; 271 Center for Data Sciences, Brigham and Women’s Hospital, Boston, MA USA; 272 Institut de Biotecnologia i de Biomedicina, Universitat Autònoma de Barcelona, Bellaterra, Barcelona, Spain; 273 Institute for Medical Microbiology and Hospital Epidemiology, Hannover Medical School, Hannover, Germany; 274 Randaberg Municipality, Randaberg, Norway; 275 Department of Quality and Health Technology, Faculty of Health Sciences, University of Stavanger, Stavanger, Norway; 276 University Hospital Schleswig-Holstein, Campus Kiel, Kiel, Germany; 277 Research Department, Stavanger University Hospital, Stavanger, Norway; 278 Department of Genetics and Bioinformatics (HDGB), Division of Health Data and Digitalization, Norwegian Institute of Public Health, Oslo, Norway; 279 Centre for Genetics and Genomics Versus Arthritis, Centre for Musculoskeletal Research, Manchester Academic Health Science Centre, The University of Manchester, Manchester, UK; 280 Department of Intensive Care, Hospital Universitario Ramón y Cajal, Instituto Ramón y Cajal de Investigación Sanitaria (IRYCIS), University of Alcalá, Madrid, Spain; 281 Clinical Biochemistry Department, Donostialdea Integrated Health Organisation, Osakidetza Basque Health Service, San Sebastian, Spain; 282 Research Center and Memory Clinic, Ace Alzheimer Center Barcelona—Universitat Internacional de Catalunya, Barcelona, Spain; 283 CIBERNED, Network Center for Biomedical Research in Neurodegenerative Diseases, National Institute of Health Carlos III, Madrid, Spain; 284 Department of Biochemistry, University Hospital Vall d’Hebron, Barcelona, Spain; 285 Department of Acute Medicine, Oslo University Hospital, Oslo, Norway; 286 Division of Neurogenetics and Molecular Psychiatry, Department of Psychiatry and Psychotherapy, Faculty of Medicine and University Hospital Cologne, University of Cologne, Cologne, Germany; 287 Department of Neurodegenerative Diseases and Geriatric Psychiatry, Medical Faculty, University Hospital Bonn, Bonn, Germany; 288 German Center for Neurodegenerative Diseases (DZNE), Bonn, Germany; 289 Department of Psychiatry, Glenn Biggs Institute for Alzheimer’s and Neurodegenerative Diseases, San Antonio, TX USA; 290 Department of Anesthesiology and Intensive Care, University Hospital of North Norway, Tromsø, Norway; 291 Pediatrics and Tettamanti Center, Fondazione IRCCS San Gerardo dei Tintori and School of Medicine and Surgery, University of Milano-Bicocca, Monza, Italy; 292 Biochemistry Department, Echevarne Laboratory, Sant Cugat del Vallés, Barcelona, Spain; 293 Centre for Multidisciplinary Research in Health Science (MACH), University of Milan, Milan, Italy; 294 Gastroenterology Unit, Fondazione IRCCS Casa Sollievo della Sofferenza, San Giovanni Rotondo, Italy; 295 Microbiology Unit, Fondazione IRCCS San Gerardo, Monza, Italy; 296 Department of Infectious Diseases, Oslo University Hospital, Oslo, Norway; 297 Humanitas Gavazzeni-Castelli, Bergamo, Italy; 298 Microbiology Department, Hospital Universitari Vall d’Hebron, Barcelona, Spain; 299 Department of Respiratory Diseases, Hospital Universitario Ramón y Cajal, Instituto Ramón y Cajal de Investigación Sanitaria (IRYCIS), University of Alcalá, Centro de Investigación Biomédica en Red en Enfermedades Respiratorias (CIBERES), Madrid, Spain; 300 Department of Respiratory Medicine and Allergology, University Hospital, Goethe University, Frankfurt am Main, Germany; 301 Centro de Investigación Biomédica en Red de Enfermedades Respiratorias (CIBERES), Madrid, Spain; 302 Centro de Investigación Biomédica en Red de Epidemiología y Salud Pública (CIBERESP), Madrid, Spain; 303 Consejo Superior de Investigaciones Científicas, Seville, Spain; 304 Department of Infectious Diseases, Hospital Universitario Ramón y Cajal, Instituto Ramón y Cajal de Investigación Sanitaria (IRYCIS), University of Alcalá, Madrid, Spain; 305 Department of Transfusion Medicine and Haematology Laboratory, San Gerardo Hospital, Monza, Italy; 306 Respiratory Medicine & International Health, University of Lübeck, Lübeck, Germany; 307 Division of Clinical Infectious Diseases, Research Center Borstel, Borstel, Germany; 308 Clinical Tuberculosis Unit, German Center for Infection Research (DZIF), Borstel, Germany; 309 Internal Medicine Department, Fondazione IRCCS Ca’ Granda Ospedale Maggiore Policlinico, Milan, Italy; 310 Humanitas Clinical and Research Center, IRCCS, Milan, Italy; 311 Respiratory Service, Basurto University Hospital, Osakidetza Basque Health Service, Bilbao, Spain; 312 Department of Clinical and Molecular Medicine, Faculty of Medicine and Health Sciences, Norwegian University of Science and Technology, Trondheim, Norway; 313 Department of Medicine, Møre & Romsdal Hospital Trust, Ålesund, Norway; 314 Department of Internal Medicine II, Medical University of Innsbruck, Innsbruck, Austria; 315 Department of Anesthesiology and Critical Care, Hospital Universitario Ramón y Cajal, Instituto Ramón y Cajal de Investigación Sanitaria (IRYCIS), University of Alcalá, Madrid, Spain; 316 Immunohematology Department, Banc de Sang i Teixits, Autonomous University of Barcelona, Barcelona, Spain; 317 European Reference Network on Hepatological Diseases (ERN RARE-LIVER), San Gerardo Hospital, Monza, Italy; 318 Department of Medical Sciences, Università degli Studi di Torino, Turin, Italy; 319 Respiratory Service, Galdakao Hospital, Osakidetza Basque Health Service, Galdakao, Spain; 320 Biocruces Bizkaia Health Research Institute, Barakaldo, Spain; 321 Department of Infectious Diseases, E.O. Ospedali Galliera, Genova, Italy; 322 Accident & Emergency and Emergency Medicine Unit, San Gerardo Hospital, Monza, Italy; 323 Histocompatibilidad y Biologia Molecular, Centro de Transfusion de Madrid, Madrid, Spain; 324 Dino Ferrari Center, Department of Pathophysiology and Transplantation, University of Milan, Milan, Italy; 325 Neurology Unit, IRCCS Fondazione Ca’ Granda Ospedale Maggiore Policlinico, Milan, Italy; 326 Department of Pathophysiology and Transplantation, University of Milan, Milan, Italy; 327 Angelo Bianchi Bonomi Hemophilia and Thrombosis Center, Fondazione IRCCS Ca’ Granda Ospedale Maggiore Policlinico, Milan, Italy; 328 Respiratory Unit, Fondazione IRCCS Ca’ Granda Ospedale Maggiore Policlinico, Milan, Italy; 329 Department of Pathophysiology and Transplantation, Università degli Studi di Milano, Milan, Italy; 330 Internal Medicine Department, Virgen del Rocio University Hospital, Seville, Spain; 331 Biochemistry Department, University Hospital Vall d’Hebron, Barcelona, Spain; 332 Centre for Medical Sciences-CISMed, University of Trento, Trento, Italy; 333 Anesthesia and Intensive Care, Santa Chiara Hospital, APSS Trento, Trento, Italy; 334 Department of Anesthesiology, Intensive Care and Emergency, Fondazione IRCCS Ca’ Granda Ospedale Maggiore Policlinico, Milan, Italy; 335 Fondazione Grigioni per il Morbo di Parkinson and Parkinson Institute, ASST Gaetano Pini-CTO, Milan, Italy; 336 School of Medicine and Surgery, University of Milano-Bicocca, Milan, Italy; 337 Acute Geriatric Unit, IRCCS San Gerardo Foundation, Monza, Italy; 338 Neurointensive Care Unit, IRCCS San Gerardo dei Tintori, Monza, Italy; 339 Emergency Department, Anesthesia and Intensive Care, IRCCS San Gerardo dei Tintori, Monza, Italy; 340 Department of Anesthesiology, Intensive Care Medicine and Pain Therapy, University Hospital Frankfurt, Frankfurt am Main, Germany; 341 Acute Geriatric Unit, IRCCS San Gerardo Foundation Monza, Monza, Italy; 342 Department of Medical Microbiology, Clinic of Laboratory Medicine, St Olavs Hospital, Trondheim, Norway; 343 Department of Internal Medicine I, University Medical Center Schleswig-Holstein, Kiel, Germany; 344 Department of Infectious Diseases, St Olavs Hospital, Trondheim University Hospital, Trondheim, Norway; 345 Department of Clinical and Molecular Medicine, NTNU, Trondheim, Norway; 346 Institute of Parasitology and Biomedicine Lopez-Neyra, CSIC, Granada, Spain; 347 Institute for Cardiogenetics, University of Lübeck, Lübeck, Germany; 348 German Research Center for Cardiovascular Research, partner site Hamburg–Lübeck–Kiel, Lübeck, Germany; 349 University Heart Center Lübeck, Lübeck, Germany; 350 Respiratory ICU, Institut Clínic Respiratory, Hospital Clinic, University of Barcelona, and IDIBAPS, Barcelona, Spain; 351 Bioinformatics Area, Fundación Progreso y Salud and Institute of Biomedicine of Sevilla (IBIS), Seville, Spain; 352 Department of Research, Ostfold Hospital Trust, Gralum, Norway; 353 Department of Infectious Diseases, Hospital Universitario Clinico San Cecilio, Granada, Spain; 354 Infectious Diseases Service, Osakidetza, Biocruces Bizkaia Health Research Institute, Barakaldo, Spain; 355 Department of Research, St Olav Hospital, Trondheim University Hospital, Trondheim, Norway; 356 Novo Nordisk Foundation Center for Protein Research, Disease Systems Biology, Faculty of Health and Medical Sciences, University of Copenhagen, Copenhagen, Denmark; 357 Medical Department, Drammen Hospital, Vestre Viken Hospital Trust, Gjettum, Norway; 358 Research Center Borstel, BioMaterialBank Nord, Sülfeld, Germany; 359 German Center for Lung Research (DZL), Airway Research Center North (ARCN), Grosshansdorf, Germany; 360 Popgen 2.0 Network (P2N), Kiel, Germany; 361 Department of Clinical Science, University of Bergen, Bergen, Norway; 362 Pediatric Departement and Centro Tettamanti—European Reference Network (ERN) PaedCan, EuroBloodNet, MetabERN-University of Milano-Bicocca-Fondazione MBBM/Ospedale San Gerardo, San Gerardo, Italy; 363 Germans Trias i Pujol Research Institute (IGTP), Badalona, Spain; 364 Clinic of Medicine and Rehabilitation, Levanger Hospital, Nord-Trondelag Hospital Trust, Levanger, Norway; 365 Geminicenter for Sepsis Research, Institute of Circulation and Medical Imaging (ISB), NTNU, Trondheim, Norway; 366 HLA Laboratory, E.O. Ospedali Galliera, Genova, Italy; 367 ISGlobal, Barcelona, Spain; 368 Universitat Pompeu Fabra (UPF), Barcelona, Spain; 369 IMIM (Hospital del Mar Medical Research Institute), Barcelona, Spain; 370 Stefan-Morsch-Stiftung, Birkenfeld, Germany; 371 Osakidetza, OSI Donostialdea, Altza Primary Care, Biodonostia Health Research Institute, San Sebastián, Spain; 372 Center of Bioinformatics, Biostatistics and Bioimaging, School of Medicine and Surgery, University of Milano-Bicocca, Milan, Italy; 373 Biostatistics and Clinical Epidemiology, Fondazione IRCCS San Gerardo dei Tintori, Monza, Italy; 374 Department of Internal Medicine, Infectious Diseases, University Hospital Frankfurt & Goethe University Frankfurt, Frankfurt am Main, Germany; 375 Phase 1 Research Centre, ASST Monza, School of Medicine and Surgery, University of Milano-Bicocca, Milan, Italy; 376 Division of Intensive Care and Emergency Medicine, Department of Internal Medicine, Medical University Innsbruck, Innsbruck, Austria; 377 Department of Anesthesiology, Hospital Universitario Ramón y Cajal, Instituto Ramón y Cajal de Investigación Sanitaria (IRYCIS), Madrid, Spain; 378 University of Cologne, Faculty of Medicine and University Hospital Cologne, Clinical Trials Centre Cologne (ZKS Köln), Cologne, Germany; 379 Pulmonology Unit, Fondazione IRCCS San Gerardo dei Tintori, Monza, Italy; 380 Infectious Diseases Unit, Fondazione IRCCS San Gerardo dei Tintori, Monza, Italy; 381 Institute of Human Genetics, University of Bonn School of Medicine & University Hospital Bonn, Bonn, Germany; 382 Institute of Immunology, Christian-Albrechts-University of Kiel & UKSH Schleswig-Holstein, Kiel, Germany; 383 Intensive Care Department, Vall d’Hebron University Hospital, SODIR-VHIR research group, Barcelona, Spain; 384 Department of Medicine, Møre & Romsdal Hospital Trust, Molde, Norway; 385 Institute of Medical Virology, University Hospital Frankfurt, Goethe University, Frankfurt am Main, Germany; 386 German Centre for Infection Research (DZIF), external partner site Frankfurt, Frankfurt am Main, Germany; 387 Department of Neurology, Bezirksklinikum Regensburg, University of Regensburg, Regensburg, Germany; 388 Institute of Transfusionsmedicine, University Hospital Schleswig-Holstein (UKSH), Kiel, Germany; 389 School of Biological Sciences, Monash University, Clayton, Victoria Australia; 390 Department of Microbiology, University Hospital Vall d’Hebron, Barcelona, Spain; 391 Autonoma University of Barcelona, Barcelona, Spain; 392 Biochemistry Unit, Hospital Universitario Clinico San Cecilio, Granada, Spain; 393 Department of Infectious Diseases, University Hospital of North Norway, Tromsø, Norway; 394 Faculty of Health Sciences, UIT The Arctic University of Norway, Tromsø, Norway; 395 Catalan Institute of Oncology (ICO), Barcelona, Spain; 396 Bellvitge Biomedical Research Institute (IDIBELL), Barcelona, Spain; 397 Universitat de Barcelona (UB), Barcelona, Spain; 398 Faculty of Medicine and University Hospital Cologne, Institute for Transfusion Medicine, University of Cologne, Cologne, Germany; 399 Departamento de Anatomía Patológica, Facultad de Medicina, Universidad de Chile, Santiago, Chile; 400 Servicio de Anatomía Patológica, Hospital Clínico de la Universidad de Chile, Santiago, Chile; 401 Laboratorio Clínico del Área Técnica de Biología Molecular, Hospital del Salvador, Providencia, Chile; 402 Departamento de Biotecnología, Universidad de Antofagasta, Antofagasta, Chile; 403 AUSTRAL-omics, Vicerrectoría de Investigación, Desarrollo y Creación Artística, Antofagasta, Chile; 404 Instituto de Ciencias Ambientales y Evolutivas, Facultad de Ciencias, Universidad Austral de Chile, Valdivia, Chile; 405 Escuela de Medicina, Universidad de Magallanes, Punta Arenas, Chile; 406 Genómica Evolutiva y Médica de Magallanes (GEMMa), Centro Asistencial, Docente y de Investigación (CADI-UMAG), Punta Arenas, Chile; 407 Interuniversity Center on Healthy Aging, Punta Arenas, Chile; 408 Instituto de Investigación Interdisciplinaria, Universidad de Talca, Talca, Chile; 409 Programa de Genética Humana, ICBM, Universidad de Chile, Santiago, Chile; 410 Departamento de Oncología Básico Clínica, Universidad de Chile, Santiago, Chile; 411 Departamento de Ciencias de la Computación, Facultadad de Ciencias Físicas y Matemáticas, Universidad de Chile, Santiago, Chile; 412 Laboratory of Chemical Carcinogenesis and Pharmacogenetics, Department of Basic and Clinical Oncology (DOBC), Faculty of Medicine, University of Chile, Santiago, Chile; 413 Latin American Network for Implementation and Validation of Clinical Pharmacogenomics Guidelines (RELIVAF-CYTED), Santiago, Chile; 414 Department of Pharmaceutical Sciences and Technology, Faculty of Chemical and Pharmaceutical Sciences, University of Chile, Santiago, Chile; 415 Department of Basic and Clinical Oncology (DOBC), Faculty of Medicine, University of Chile, Santiago, Chile; 416 University of Chile & Latin American Network for Implementation and Validation of Clinical Pharmacogenomics Guidelines (RELIVAF-CYTED), Santiago, Chile; 417 Departamento de Tecnología Médica, Facultad de Ciencias de la Salud, Universidad de Antofagasta, Antofagasta, Chile; 418 AUSTRAL-omics, Vicerrectoría de Investigación, Desarrollo y Creación Artística, Universidad Austral de Chile, Valdivia, Chile; 419 Clinical Biochemistry and Immunology Department, Faculty of Pharmacy, Universidad de Concepción, Concepción, Chile; 420 Department of Clinical Immunology, Zealand University Hospital, Køge, Denmark; 421 Department of Epidemiology Research, Statens Serum Institut, Copenhagen, Denmark; 422 Department of Clinical Immunology, Rigshospitalet, University of Copenhagen, Copenhagen, Denmark; 423 Department of Infectious Diseases, Copenhagen University Hospital (Rigshospitalet), Copenhagen, Denmark; 424 Department of Medical Endocrinology and Metabolism, Copenhagen University Hospital (Rigshospitalet), Copenhagen, Denmark; 425 Department of Hematology, Rigshospitalet, Copenhagen, Denmark; 426 Statens Serum Institut, Copenhagen, Denmark; 427 Department of Pediatrics, Faculty of Medicine, Mansoura University, Mansoura, Egypt; 428 Department of Surgery, Faculty of Medicine, Mansoura University, Mansoura, Egypt; 429 Stanley Center for Psychiatric Research & Program in Medical and Population Genetics, Broad Institute of MIT and Harvard, Cambridge, MA USA; 430 Chest Department, Faculty of Medicine, Mansoura University, Mansoura, Egypt; 431 Anesthesia, Surgical Intensive Care & Pain Management Department, Faculty of Medicine, Tanta University, Tanta, Egypt; 432 Department of Medical Biochemistry, Faculty of Medicine, Mansoura University, Mansoura, Egypt; 433 Department of Tropical Medicine, Faculty of Medicine, Mansoura University, Mansoura, Egypt; 434 Pediatric and Neonatology, Kafr Elzayat General Hospital, Kafr El-Zayat, Egypt; 435 Chest Department, Faculty of Medicine, Tanta University, Tanta, Egypt; 436 Pediatrics Department, Faculty of Medicine, Tanta University, Tanta, Egypt; 437 Anesthesia, Surgical Intensive Care & Pain Management Departmen, Faculty of Medicine, Mansoura University, Mansoura, Egypt; 438 Department of Population Health and Reproduction, School of Veterinary Medicine, University of California, Davis, Davis, CA USA; 439 Department of Anaethesia and Critical Care, Faculty of Medicine, Mansoura University, Mansoura, Egypt; 440 Department of Internal Medicine, Faculty of Medicine, Tanta University, Tanta, Egypt; 441 Faculty of Science, Tanta University, Tanta, Egypt; 442 Department of Internal Medicine, Erasmus MC, University Medical Center, Rotterdam, The Netherlands; 443 Department of Epidemiology, Erasmus MC, University Medical Center, Rotterdam, The Netherlands; 444 Department of Internal Medicine, Erasmus MC, Rotterdam, The Netherlands; 445 FinnGen, Helsinki, Finland; 446 Institute for Molecular Medicine Finland (FIMM), HiLIFE, University of Helsinki, Helsinki, Finland; 447 Public Health, Faculty of Medicine, University of Helsinki, Helsinki, Finland; 448 Finnish Institute for Health and Welfare (THL), Helsinki, Finland; 449 Clinical and Molecular Metabolism Research Program, Faculty of Medicine, University of Helsinki, Helsinki, Finland; 450 Department of Computational Biology, Institut Pasteur, Université de Paris, Paris, France; 451 Université Paris-Saclay, CEA, Centre National de Recherche en Génomique Humaine (CNRGH), Evry, France; 452 University of Bordeaux, INSERM, Bordeaux Population Health Center, UMR1219, Bordeaux, France; 453 Department of Neurology, Institute of Neurodegenerative Diseases, Bordeaux University Hospital, Bordeaux, France; 454 Laboratory of Human Genetics of Infectious Diseases, Necker Branch, University of Paris, INSERM U1163, Imagine Institute, Paris, France; 455 St Giles Laboratory of Human Genetics of Infectious Diseases, Rockefeller Branch, The Rockefeller University, New York, NY USA; 456 Department of Global Health, Institut Pasteur, Université de Paris, Paris, France; 457 Department of Nephrology, Transplantation, Dialysis and Apheresis, Bordeaux University Hospital, Bordeaux, France; 458 Immunoconcept CNRS UMR 5164, Bordeaux University, Bordeaux, France; 459 St Giles Laboratory of Human Genetics of Infectious Diseases, Rockefeller Branch, The Rockefeller University, New York, NY USA; 460 Université de Paris, INSERM, IAME UMR 1137, Paris, France; 461 UMR-CNRS 6249 Chrono-environnement, Université Bourgogne Franche-Comté, Besançon, France; 462 Human Evolutionary Genetics, Institut Pasteur, UMR 2000, CNRS, Paris, France; 463 Chair of Human Genomics and Evolution, Collège de France, Paris, France; 464 Human Evolutionary Genetics Unit, Institut Pasteur, Université Paris Cité, CNRS UMR2000, Paris, France; 465 Translational Immunology Laboratory, Institut Pasteur, Paris, France; 466 Catalan Institute of Oncology, Bellvitge Biomedical Research Institute, Consortium for Biomedical Research in Epidemiology and Public Health and University of Barcelona, Barcelona, Spain; 467 CIBER Epidemiología y Salud Pública (CIBERESP), Madrid, Spain; 468 Barcelona Supercomputing Center—Centro Nacional de Supercomputación (BSC-CNS), Life & Medical Sciences, Barcelona, Spain; 469 Programs in Metabolism and Medical and Population Genetics, Broad Institute of MIT and Harvard, Cambridge, MA USA; 470 Department of Medicine, Harvard Medical School, Boston, MA USA; 471 Diabetes Unit and Center for Genomic Medicine, Massachusetts General Hospital, Boston, MA USA; 472 CIBER de Enfermedades Infecciosas (CIBERINFEC), Barcelona, Spain; 473 Blizard Institute, Queen Mary University of London, London, UK; 474 School of Basic and Medical Biosciences, Faculty of Life Sciences and Medicine, King’s College London, London, UK; 475 Wolfson Institute of Population Health, Queen Mary University of London, London, UK; 476 Medical and Population Genomics, Wellcome Sanger Institute, Hinxton, UK; 477 Bradford Institute for Health Research, Bradford Teaching Hospitals National Health Service (NHS) Foundation Trust, Bradford, UK; 478 Genes & Health, Blizard Institute, Queen Mary University of London, London, UK; 479 Genentech, San Francisco, CA USA; 480 DNA Link, Seoul, Republic of Korea; 481 Seoul National University Hospital Gangnam Center, Seoul, Republic of Korea; 482 Division of Infectious Diseases, Department of Internal Medicine, Chungnam National University School of Medicine, Daejeon, Republic of Korea; 483 Department of Internal Medicine, School of Medicine, Kyungpook National University, Daegu, Republic of Korea; 484 Division of Infectious Diseases, Department of Internal Medicine, Incheon Medical Center, Incheon, Republic of Korea; 485 Department of Infectious Diseases, Keimyung University Dongsan Hospital, Keimyung University School of Medicine, Daegu, Republic of Korea; 486 Department of Internal Medicine, Pusan National University School of Medicine and Medical Research Institute, Pusan National University Hospital, Busan, Republic of Korea; 487 Division of Infectious Diseases, Department of Internal Medicine, Myongji Hospital, Goyang, Republic of Korea; 488 Institute for Health Promotion, Graduate School of Public Health, Yonsei University, Seoul, Republic of Korea; 489 Department of Precision Medicine, National Institute of Health, Cheongwon-gun, Republic of Korea; 490 Division of Genome Science, Department of Precision Medicine, National Institute of Health, Cheongwon-gun, Republic of Korea; 491 Baillie Gifford Pandemic Science Hub, Centre for Inflammation Research, The Queen’s Medical Research Institute, University of Edinburgh, Edinburgh, UK; 492 MRC Human Genetics Unit, Institute of Genetics and Cancer, University of Edinburgh, Western General Hospital, Edinburgh, UK; 493 Roslin Institute, University of Edinburgh, Easter Bush, Edinburgh, UK; 494 William Harvey Research Institute, Queen Mary University of London, London, UK; 495 Intensive Care Unit, Royal Infirmary of Edinburgh, Edinburgh, UK; 496 Pain Service, NHS Tayside, Ninewells Hospital and Medical School, Dundee, UK; 497 Centre for Inflammation Research, The Queen’s Medical Research Institute, University of Edinburgh, Edinburgh, UK; 498 Wellcome Centre for Human Genetics, University of Oxford, Oxford, UK; 499 Institute for Molecular Bioscience, The University of Queensland, Brisbane, Queensland, Australia; 500 Biostatistics Group, Greater Bay Area Institute of Precision Medicine (Guangzhou), Fudan University, Guangzhou, China; 501 Centre for Global Health Research, Usher Institute of Population Health Sciences and Informatics, Edinburgh, UK; 502 Edinburgh Clinical Research Facility, Western General Hospital, University of Edinburgh, Edinburgh, UK; 503 School of Life Sciences, Westlake University, Hangzhou, China; 504 Westlake Laboratory of Life Sciences and Biomedicine, Hangzhou, China; 505 Great Ormond Street Hospital, London, UK; 506 Royal Hospital for Children, Glasgow, UK; 507 Centre for Tropical Medicine and Global Health, Nuffield Department of Medicine, University of Oxford, Oxford, UK; 508 Department of Anaesthesia and Intensive Care, The Chinese University of Hong Kong, Prince of Wales Hospital, Hong Kong, China; 509 Department of Critical Care Medicine, Queen’s University and Kingston Health Sciences Centre, Kingston, Ontario Canada; 510 Wellcome-Wolfson Institute for Experimental Medicine, Queen’s University Belfast, Belfast, UK; 511 Department of Intensive Care Medicine, Royal Victoria Hospital, Belfast, UK; 512 UCL Centre for Human Health and Performance, London, UK; 513 Clinical Research Centre at St Vincent’s University Hospital, University College Dublin, Dublin, Ireland; 514 National Heart and Lung Institute, Imperial College London, London, UK; 515 Imperial College Healthcare NHS Trust: London, London, UK; 516 Heart Institute, University of Sao Paulo, Sao Paulo, Brazil; 517 Intensive Care National Audit & Research Centre, London, UK; 518 NIHR Health Protection Research Unit for Emerging and Zoonotic Infections, Institute of Infection, Veterinary and Ecological Sciences, University of Liverpool, Liverpool, UK; 519 Respiratory Medicine, Alder Hey Children’s Hospital, Institute in The Park, University of Liverpool, Liverpool, UK; 520 Department of Medicine, University of Cambridge, Cambridge, UK; 521 Guys and St Thomas’ Hospital, London, UK; 522 James Cook University Hospital, Middlesbrough, UK; 523 Barts Health NHS Trust, London, UK; 524 Royal Stoke University Hospital, Stoke-on-Trent, UK; 525 North Middlesex University Hospital NHS trust, London, UK; 526 King’s College Hospital, London, UK; 527 Charing Cross Hospital, St Mary’s Hospital and Hammersmith Hospital, London, UK; 528 The Royal Liverpool University Hospital, Liverpool, UK; 529 John Radcliffe Hospital, Oxford, UK; 530 Addenbrooke’s Hospital, Cambridge, UK; 531 Nottingham University Hospital, Nottingham, UK; 532 St George’s Hospital, London, UK; 533 BHRUT (Barking Havering)—Queens Hospital and King George Hospital, London, UK; 534 Royal Infirmary of Edinburgh, Edinburgh, UK; 535 Kingston Hospital, London, UK; 536 Queen Alexandra Hospital, Portsmouth, UK; 537 Royal Gwent Hospital, Newport, UK; 538 Royal Blackburn Teaching Hospital, Blackburn, UK; 539 Stepping Hill Hospital, Stockport, UK; 540 Northumbria Healthcare NHS Foundation Trust, North Shields, UK; 541 Countess of Chester Hospital, Chester, UK; 542 Pinderfields General Hospital, Wakefield, UK; 543 Ninewells Hospital, Dundee, UK; 544 Croydon University Hospital, Croydon, UK; 545 Morriston Hospital, Swansea, UK; 546 Queen Elizabeth University Hospital, Glasgow, UK; 547 Broomfield Hospital, Chelmsford, UK; 548 Heartlands Hospital, Birmingham, UK; 549 Royal Sussex County Hospital, Brighton, UK; 550 York Hospital, York, UK; 551 Queen Elizabeth Hospital, Birmingham, UK; 552 Royal Glamorgan Hospital, Pontyclun, UK; 553 Barnet Hospital, London, UK; 554 Wythenshawe Hospital, Manchester, UK; 555 Medway Maritime Hospital, Gillingham, UK; 556 Royal Berkshire NHS Foundation Trust, Reading, UK; 557 Whiston Hospital, Prescot, UK; 558 The Royal Oldham Hospital, Manchester, UK; 559 Chesterfield Royal Hospital Foundation Trust, Chesterfield, UK; 560 Aberdeen Royal Infirmary, Aberdeen, UK; 561 Royal Devon and Exeter Hospital, Exeter, UK; 562 Glasgow Royal Infirmary, Glasgow, UK; 563 Blackpool Victoria Hospital, Blackpool, UK; 564 Southampton General Hospital, Southampton, UK; 565 Ashford and St Peter’s Hospital, Chertsey, UK; 566 Derriford Hospital, Plymouth, UK; 567 East Surrey Hospital, Redhill, UK; 568 Poole Hospital, Poole, UK; 569 Royal Alexandra Hospital, Paisley, UK; 570 St James’s University Hospital and Leeds General Infirmary, Leeds, UK; 571 Bedford Hospital, Bedford, UK; 572 Southport and Formby District General Hospital, Ormskirk, UK; 573 The Tunbridge Wells Hospital and Maidstone Hospital, Kent, UK; 574 Queen Elizabeth Hospital, Woolwich, London, UK; 575 North Manchester General Hospital, Manchester, UK; 576 Royal Victoria Infirmary, Newcastle Upon Tyne, UK; 577 Hull Royal Infirmary, Hull, UK; 578 Manchester Royal Infirmary, Manchester, UK; 579 Royal Derby Hospital, Derby, UK; 580 Aintree University Hospital, Liverpool, UK; 581 Fairfield General Hospital, Bury, UK; 582 Norfolk and Norwich University hospital (NNUH), Norwich, UK; 583 Milton Keynes University Hospital, Milton Keynes, UK; 584 Good Hope Hospital, Birmingham, UK; 585 Queen Elizabeth Hospital Gateshead, Gateshead, UK; 586 Royal Bolton Hospital, Bolton, UK; 587 Tameside General Hospital, Ashton Under Lyne, UK; 588 Salford Royal Hospital, Manchester, UK; 589 Great Ormond St Hospital and UCL Great Ormond St Institute of Child Health NIHR Biomedical Research Centre, London, UK; 590 Southmead Hospital, Bristol, UK; 591 William Harvey Hospital, Ashford, UK; 592 Arrowe Park Hospital, Wirral, UK; 593 Royal Hampshire County Hospital, Winchester, UK; 594 Bradford Royal Infirmary, Bradford, UK; 595 Glan Clwyd Hospital, Bodelwyddan, UK; 596 Royal Bournemouth Hospital, Bournemouth, UK; 597 Bristol Royal Infirmary, Bristol, UK; 598 University Hospital North Durham, Darlington, UK; 599 Darlington Memorial Hospital, Darlington, UK; 600 Basildon Hospital, Basildon, UK; 601 University College Hospital, London, UK; 602 Whittington Hospital, London, UK; 603 Western General Hospital, Edinburgh, UK; 604 Ipswich Hospital, Ipswich, UK; 605 Hereford County Hospital, Hereford, UK; 606 Sunderland Royal Hospital, Sunderland, UK; 607 Queens Hospital Burton, Burton-On-Trent, UK; 608 Musgrove Park Hospital, Taunton, UK; 609 The Royal Papworth Hospital, Cambridge, UK; 610 University Hospital Lewisham, London, UK; 611 The Princess Alexandra Hospital, Harlow, UK; 612 University Hospital of Wales, Cardiff, UK; 613 West Middlesex Hospital, Isleworth, UK; 614 Royal Albert Edward Infirmary, Wigan, UK; 615 Stoke Mandeville Hospital, Aylesbury, UK; 616 Royal Lancaster Infirmary, Lancaster, UK; 617 Basingstoke and North Hampshire Hospital, Basingstoke, UK; 618 Worthing Hospital, Worthing, UK; 619 St Richard’s Hospital, Chichester, UK; 620 The Alexandra Hospital, Redditch and Worcester Royal Hospital, Worcester, UK; 621 Royal Cornwall Hospital, Truro, UK; 622 Watford General Hospital, Watford, UK; 623 Macclesfield District General Hospital, Macclesfield, UK; 624 Royal Surrey County Hospital, Guildford, UK; 625 Rotherham General Hospital, Rotherham, UK; 626 Craigavon Area Hospital, Craigavon, UK; 627 King’s Mill Hospital, Nottingham, UK; 628 Dumfries and Galloway Royal Infirmary, Dumfries, UK; 629 Prince Charles Hospital, Merthyr Tydfil, UK; 630 Ysbyty Gwynedd, Bangor, UK; 631 Royal Preston Hospital, Preston, UK; 632 The Great Western Hospital, Swindon, UK; 633 Lincoln County Hospital, Lincoln, UK; 634 University Hospital of North Tees, Stockton on Tees, UK; 635 Glangwili General Hospital, Camarthen, UK; 636 Southend University Hospital, Westcliff-on-Sea, UK; 637 Lister Hospital, Stevenage, UK; 638 Diana Princess of Wales Hospital, Grimsby, UK; 639 West Suffolk Hospital, Bury St Edmunds, UK; 640 Victoria Hospital, Kirkcaldy, UK; 641 Calderdale Royal Hospital, Halifax, UK; 642 Huddersfield Royal Infirmary, Huddersfield, UK; 643 Dorset County Hospital, Dorchester, UK; 644 Russell’s Hall Hospital, Dudley, UK; 645 Royal United Hospital, Bath, UK; 646 St Mary’s Hospital, Newport, UK; 647 George Eliot Hospital NHS Trust, Nuneaton, UK; 648 Yeovil Hospital, Yeovil, UK; 649 Forth Valley Royal Hospital, Falkirk, UK; 650 Frimley Park Hospital, Camberley, UK; 651 Chelsea & Westminster NHS Foundation Trust, London, UK; 652 Queen Elizabeth the Queen Mother Hospital, Margate, UK; 653 Royal Brompton Hospital, London, UK; 654 Darent Valley Hospital, Dartford, UK; 655 University Hospital Crosshouse, Kilmarnock, UK; 656 University Hospital Wishaw, Wishaw, UK; 657 University College Dublin, St Vincent’s University Hospital, Dublin, Ireland; 658 The Queen Elizabeth Hospital, King’s Lynn, UK; 659 Walsall Manor Hospital, Walsall, UK; 660 Princess Royal Hospital, Brighton, UK; 661 Barnsley Hospital, Barnsley, UK; 662 Warrington General Hospital, Warrington, UK; 663 Royal Victoria Hospital, Belfast, UK; 664 Royal Hallamshire Hospital and Northern General Hospital, Sheffield, UK; 665 Harefield Hospital, London, UK; 666 Cumberland Infirmary, Carlisle, UK; 667 Eastbourne District General Hospital, Eastbourne, UK; 668 Conquest Hospital, Saint Leonards-on-sea, UK; 669 Salisbury District Hospital, Salisbury, UK; 670 Airedale General Hospital, Keighley, UK; 671 Leicester Royal Infirmary, Leicester, UK; 672 Peterborough City Hospital, Peterborough, UK; 673 Hinchingbrooke Hospital, Huntingdon, UK; 674 Colchester General Hospital, Colchester, UK; 675 Princess Royal Hospital, Telford and Royal Shrewsbury Hospital, Shrewsbury, UK; 676 University Hospital Monklands, Airdrie, UK; 677 Wrexham Maelor Hospital, Wrexham, UK; 678 New Cross Hospital, Wolverhampton, UK; 679 University Hospital Hairmyres, East Kilbride, UK; 680 Warwick Hospital, Warwick, UK; 681 Sandwell General Hospital and City Hospital, Birmingham, UK; 682 Royal Manchester Children’s Hospital, Manchester, UK; 683 Gloucestershire Royal Hospital, Gloucester, UK; 684 University Hospitals Coventry & Warwickshire NHS Trust, Coventry, UK; 685 Torbay Hospital, Torquay, UK; 686 Pilgrim Hospital, Lincoln, UK; 687 Prince Philip Hospital, Lianelli, UK; 688 Princess of Wales Hospital, Llantrisant, UK; 689 Northampton General Hospital NHS Trust, Northampton, UK; 690 The Christie NHS Foundation Trust, Manchester, UK; 691 James Paget University Hospital NHS Trust, Great Yarmouth, UK; 692 Birmingham Children’s Hospital, Birmingham, UK; 693 Withybush General Hospital, Haverfordwest, UK; 694 Northwick Park Hospital, London, UK; 695 North Devon District Hospital, Barnstaple, UK; 696 Scunthorpe General Hospital, Scunthorpe, UK; 697 Royal Free Hospital, London, UK; 698 Raigmore Hospital, Inverness, UK; 699 West Cumberland Hospital, Whitehaven, UK; 700 Furness General Hospital, Barrow-in-Furness, UK; 701 Liverpool Heart and Chest Hospital, Liverpool, UK; 702 Scarborough General Hospital, Scarborough, UK; 703 Bronglais General Hospital, Aberystwyth, UK; 704 Alder Hey Children’s Hospital, Liverpool, UK; 705 Borders General Hospital, Melrose, UK; 706 Leighton Hospital, Cheshire, UK; 707 Kent & Canterbury Hospital, Canterbury, UK; 708 Harrogate and District NHS Foundation Trust, Harrogate, UK; 709 The Royal Marsden Hospital, London, UK; 710 Ealing Hospital, Southall, UK; 711 St John’s Hospital Livingston, Livingston, UK; 712 Wexham Park Hospital, Slough, UK; 713 Sheffield Children’s Hospital, Sheffield, UK; 714 Homerton University Hospital Foundation NHS Trust, London, UK; 715 National Hospital for Neurology and Neurosurgery, London, UK; 716 The Royal Alexandra Children’s Hospital, Brighton, UK; 717 Golden Jubilee National Hospital, Clydebank, UK; 718 Illumina Cambridge, Cambridge, UK; 719 Test and Trace, The Health Security Agency, Department of Health and Social Care, London, UK; 720 Imperial College London, London, UK; 721 NIHR Clinical Research Network (CRN), North West London Core Team, London, UK; 722 Cambridge University Hospitals NHS Foundation Trust, Cambridge, UK; 723 Biostatistics Group, State Key Laboratory of Biocontrol, School of Life Sciences, Sun Yat-sen University, Guangzhou, China; 724 Department of Infectious Diseases, Leiden University Medical Center, Leiden, The Netherlands; 725 Genotek, Moscow, Russia; 726 Helix, San Mateo, CA USA; 727 Center for Genomic Medicine, Desert Research Institute, Reno, NV USA; 728 Renown Health, Reno, NV USA; 729 Gujarat Biotechnology Research Centre (GBRC), Department of Science and Technology (Government of Gujarat), Gandhinagar, India; 730 Commissionerate of Health Medical Services and Medical Education Gandhinagar, Gandhinagar, India; 731 Department of Microbiology, B.J. Medical College and Civil hospital, Institute of Medical Post-Graduate Studies and Research, Ahmedabad, India; 732 Department of Medicine, B.J. Medical College and Civil hospital, Institute of Medical Post-Graduate Studies and Research, Ahmedabad, India; 733 Department of Community Medicine, Government Medical College, Surat, India; 734 Department of Microbiology, Government Medical College, Bhavnagar, India; 735 Department of General Medicine, GMERS Medical College & Hospital, Gotri, Vadodara, India; 736 Department of Community & Family Medicine, All India Institute of Medical Sciences, Rajkot, India; 737 National Laboratory of Genomics for Biodiversity (LANGEBIO), Advanced Genomics Unit (UGA), CINVESTAV, Irapuato, Guanajuato, Mexico; 738 Instituto Nacional de Enfermedades Respiratorias Ismael Cosío Villegas, Tlalpan, Mexico City, Mexico; 739 Departamento de Bioquímica, Facultad de Medicina, Universidad Nacional Autónoma de México, Coyoacán, Mexico City, Mexico; 740 Instituto Nacional de Salud Pública, Cuernavaca, Mexico; 741 International Laboratory for Human Genome Research (LIIGH), UNAM Juriquilla, Queretaro, Mexico; 742 Unidad de Biología Molecular y Medicina Genómica, Instituto Nacional de Ciencias Médicas y Nutrición Salvador Zubirán/ Instituto de Investigaciones Biomédicas, UNAM, Mexico City, Mexico; 743 Instituto Nacional de Medicina Genómica, Mexico City, Mexico; 744 Instituto Nacional de Pediatría, Mexico City, Mexico; 745 Escuela Superior de Medicina, Instituto Politécnico Nacional, Mexico City, Mexico; 746 Tecnologico de Monterrey, Escuela de Medicina y Ciencias de la Salud, Monterrey, Mexico; 747 Division of Pulmonary Medicine, Department of Medicine, Keio University School of Medicine, Tokyo, Japan; 748 Department of Statiatical Genetics, Osaka University Graduate School of Medicine, Suita, Japan; 749 Laboratory of Statistical Immunology, Immunology Frontier Research Center (WPI-IFReC), Osaka University, Suita, Japan; 750 Integrated Frontier Research for Medical Science Division, Institute for Open and Transdisciplinary Research Initiatives, Osaka University, Suita, Japan; 751 Division of Health Medical Intelligence, Human Genome Center, the Institute of Medical Science, The University of Tokyo, Tokyo, Japan; 752 Laboratory of Viral Infection I, Department of Infection Control and Immunology, Ōmura Satoshi Memorial Institute & Graduate School of Infection Control Sciences, Kitasato University, Tokyo, Japan; 753 Department of Surgery, Keio University School of Medicine, Tokyo, Japan; 754 Department of Organoid Medicine, Keio University School of Medicine, Tokyo, Japan; 755 Department of Infectious Diseases, Keio University School of Medicine, Tokyo, Japan; 756 Department of Respiratory Medicine and Clinical Immunology, Osaka University Graduate School of Medicine, Suita, Japan; 757 Department of Immunopathology, Immunology Frontier Research Center (WPI-IFReC), Osaka University, Suita, Japan; 758 Institute of Research, Tokyo Medical and Dental University, Tokyo, Japan; 759 Department of Insured Medical Care Management, Tokyo Medical and Dental University Hospital of Medicine, Tokyo, Japan; 760 Genome Medical Science Project (Toyama), National Center for Global Health and Medicine, Tokyo, Japan; 761 Division of Gastroenterology and Hepatology, Department of Medicine, Keio University School of Medicine, Tokyo, Japan; 762 M&D Data Science Center, Tokyo Medical and Dental University, Tokyo, Japan; 763 Kyoto University, Department of Pathology and Tumor Biology Institute for the Advanced Study of Human Biology (WPI-ASHBi), Kyoto University, Kyoto, Japan; 764 Department of Medicine, Center for Hematology and Regenerative Medicine, Karolinska Institute, Stockholm, Sweden; 765 Department of Emergency and Critical Care Medicine, Keio University School of Medicine, Tokyo, Japan; 766 Department of Anesthesiology, Keio University School of Medicine, Tokyo, Japan; 767 Department of Laboratory Medicine, Keio University School of Medicine, Tokyo, Japan; 768 Division of Infection Control and Prevention, Osaka University Hospital, Suita, Japan; 769 Department of Biomedical Ethics and Public Policy, Osaka University Graduate School of Medicine, Suita, Japan; 770 Center for Genomic Medicine, Kyoto University Graduate School of Medicine, Kyoto, Japan; 771 Department of Pulmonary Medicine, Faculty of Medicine, University of Tsukuba, Tsukuba, Japan; 772 Department of Neurosurgery, Faculty of Medicine, The University of Tokyo, Tokyo, Japan; 773 Laboratory of Immune Regulation, Department of Microbiology and Immunology, Osaka University Graduate School of Medicine, Suita, Japan; 774 Medical Innovation Promotion Center, Tokyo Medical and Dental University, Tokyo, Japan; 775 Clinical Research Center, Tokyo Medical and Dental University Hospital of Medicine, Tokyo, Japan; 776 Department of Medical Informatics, Tokyo Medical and Dental University Hospital of Medicine, Tokyo, Japan; 777 Respiratory Medicine, Tokyo Medical and Dental University, Tokyo, Japan; 778 Clinical Laboratory, Tokyo Medical and Dental University Hospital of Medicine, Tokyo, Japan; 779 Department of Pathology and Tumor Biology, Kyoto University, Kyoto, Japan; 780 Department of Respiratory Medicine, Juntendo University Faculty of Medicine and Graduate School of Medicine, Tokyo, Japan; 781 Department of Emergency and Disaster Medicine, Juntendo University Faculty of Medicine and Graduate School of Medicine, Tokyo, Japan; 782 Department of Cardiovascular Biology and Medicine, Juntendo University Faculty of Medicine and Graduate School of Medicine, Tokyo, Japan; 783 Department of Respiratory Medicine, Tokyo Women’s Medical University, Tokyo, Japan; 784 Department of General Medicine, Tokyo Women’s Medical University, Tokyo, Japan; 785 Department of Respiratory Medicine, Saitama Cardiovascular and Respiratory Center, Saitama, Japan; 786 Kawasaki Municipal Ida Hospital, Kawasaki, Japan; 787 Internal Medicine, Saitama Medical Center, JCHO (Japan Community Health Care Organization), Saitama, Japan; 788 Saitama City Hospital, Saitama, Japan; 789 Division of Infection Control, Eiju General Hospital, Tokyo, Japan; 790 Department of Pulmonary Medicine, Eiju General Hospital, Tokyo, Japan; 791 Department of Respiratory Medicine, Osaka Saiseikai Nakatsu Hospital, Osaka, Japan; 792 Department of Infection Control, Osaka Saiseikai Nakatsu Hospital, Osaka, Japan; 793 Department of Infectious Diseases, Tosei General Hospital, Seto, Japan; 794 Fukujuji Hospital, Kiyose, Japan; 795 Department of Emergency and Critical Care Medicine, Tokyo Women’s Medical University Medical Center East, Tokyo, Japan; 796 Department of Medicine, Tokyo Women’s Medical University Medical Center East, Tokyo, Japan; 797 Department of Pediatrics, Tokyo Women’s Medical University Medical Center East, Tokyo, Japan; 798 Japan Community Health Care Organization Kanazawa Hospital, Kanazawa, Japan; 799 Division of Pulmonary Medicine, Department of Internal Medicine, Federation of National Public Service Personnel Mutual Aid Associations, Tachikawa Hospital, Tachikawa, Japan; 800 Department of Respiratory Medicine, Japan Organization of Occupational Health and Safety, Kanto Rosai Hospital, Kawasaki, Japan; 801 Department of General Internal Medicine, Japan Organization of Occupational Health and Safety, Kanto Rosai Hospital, Kawasaki, Japan; 802 Department of Emergency and Critical Care Medicine, Kansai Medical Univrsity General Medical Center, Kirakata, Japan; 803 Department of Respiratory Medicine, Kitasato University Kitasato Institute Hospital, Tokyo, Japan; 804 Ishikawa Prefectural Central Hospital, Kanazawa, Japan; 805 Internal Medicine, Sano Kosei General Hospital, Sano, Japan; 806 Saiseikai Yokohamashi Nanbu Hospital, Yokohama, Japan; 807 Kanagawa Cardiovascular and Respiratory Center, Yokohama, Japan; 808 Saiseikai Utsunomiya Hospital, Utsunomiya, Japan; 809 Department of Respiratory Medicine, KKR Sapporo Medical Center, Sapporo, Japan; 810 Internal Medicine, Internal Medicine Center, Showa University Koto Toyosu Hospital, Tokyo, Japan; 811 Department of Respiratory Medicine, Toyohashi Municipal Hospital, Toyohashi, Japan; 812 Keiyu Hospital, Yokohama, Japan; 813 Department of Rheumatology, National Hospital Organization Hokkaido Medical Center, Sapporo, Japan; 814 Department of Respiratory Medicine, National Hospital Organization Tokyo Medical Center, Tokyo, Japan; 815 Department of Allergy, National Hospital Organization Tokyo Medical Center, Tokyo, Japan; 816 Department of General Internal Medicine and Infectious Diseases, National Hospital Organization Tokyo Medical Center, Tokyo, Japan; 817 Japanese Red Cross Musashino Hospital, Musashino, Japan; 818 Department of Respiratory Medicine, Tohoku University Graduate School of Medicine, Sendai, Japan; 819 Department of Internal Medicine, Division of Respiratory Medicine, Nihon University School of Medicine, Tokyo, Japan; 820 Department of Emergency and Critical Care Medicine, St Marianna University School of Medicine, Kawasaki, Japan; 821 Division of General Internal Medicine, Department of Internal Medicine, St Marianna University School of Medicine, Kawasaki, Japan; 822 National Hospital Organization Kanazawa Medical Center, Kanazawa, Japan; 823 Division of Infectious Diseases and Respiratory Medicine, Department of Internal Medicine, National Defense Medical College, Saitama, Japan; 824 Department of Emergency and Critical Care Medicine, Faculty of Medicine, Fukuoka University, Fukuoka, Japan; 825 Department of Infection Control, Fukuoka University Hospital, Fukuoka, Japan; 826 Tokyo Saiseikai Central Hospital, Tokyo, Japan; 827 Department of Internal Medicine, Fukuoka Tokushukai Hospital, Kasuga, Japan; 828 Department of Infectious Disease and Clinical Research Institute, National Hospital Organization Kyushu Medical Center, Fukuoka, Japan; 829 Department of Respirology, National Hospital Organization Kyushu Medical Center, Division of Respirology, Rheumatology, and Neurology, Department of Internal Medicine, Kurume University School of Medicine, Fukuoka, Japan; 830 Department of Infectious Disease, National Hospital Organization Kyushu Medical Center, Fukuoka, Japan; 831 Matsumoto City Hospital, Matsumoto, Japan; 832 Uji-Tokushukai Medical Center, Uji, Japan; 833 Department of Respiratory Medicine, Nagoya University Graduate School of Medicine, Nagoya, Japan; 834 Department of Respiratory Medicine, Fujisawa City Hospital, Fujisawa, Japan; 835 Sapporo City General Hospital, Sapporo, Japan; 836 Department of Emergency and Critical Care Medicine, Chiba University Graduate School of Medicine, Chiba, Japan; 837 Division of Respiratory Medicine, Social Welfare Organization Saiseikai Imperial Gift Foundation, Saiseikai Kumamoto Hospital, Kumamoto, Japan; 838 Department of Anesthesiology and Intensive Care Medicine, Kyoto Prefectural University of Medicine, Kyoto, Japan; 839 Ome Municipal General Hospital, Ome, Japan; 840 Hanwa Daini Hospital, Osaka, Japan; 841 Department of Respiratory Internal Medicine, St Marianna University School of Medicine, Yokohama-City Seibu Hospital, Yokohama, Japan; 842 Division of Hematology, Department of Internal Medicine, St Marianna University Yokohama-City Seibu Hospital, Yokohama, Japan; 843 Division of Pulmonary Medicine, Department of Medicine, Tokai University School of Medicine, Tokai University School of Medicine, Tokyo, Japan; 844 Division of Pulmonary Medicine, Department of Medicine, Tokai University School of Medicine, Tokyo, Japan; 845 National Hospital Organization Kumamoto Medical Center, Kumamoto, Japan; 846 Department of Respiratory Medicine, Tokyo Medical University Hospital, Tokyo, Japan; 847 Department of Respiratory Medicine, Japanese Red Cross Medical Center, Tokyo, Japan; 848 JA Toride Medical Hospital, Toride, Japan; 849 Japan Organization of Occupational Health and Safety Okayama Rosai Hospital, Okayama, Japan; 850 Emergency and Disaster Medicine, Graduate School of Medicine, Gifu University School of Medicine, Gifu, Japan; 851 Niigata University, Niigata, Japan; 852 National Hospital Organization Kyoto Medical Center, Kyoto, Japan; 853 Research Institute for Diseases of the Chest, Graduate School of Medical Sciences, Kyushu University, Fukuoka, Japan; 854 Department of Medicine and Biosystemic Science, Kyushu University Graduate School of Medical Sciences, Fukuoka, Japan; 855 Department of Emergency and Critical Care Medicine, Tsukuba University, Tsukuba, Japan; 856 Department of Nephrology, Faculty of Medicine, University of Tsukuba, Tsukuba, Japan; 857 Department of Hematology, Faculty of Medicine, University of Tsukuba, Tsukuba, Japan; 858 National Hospital Organization Tokyo Hospital, Tokyo, Japan; 859 Fujioka General Hospital, Fujioka, Japan; 860 Division of Respiratory Medicine and Allergology, Department of Medicine, School of Medicine, Showa University, Tokyo, Japan; 861 Department of Pulmonary Medicine, Fukushima Medical University, Fukushima, Japan; 862 Kansai Electric Power Hospital, Osaka, Japan; 863 Kumamoto City Hospital, Kumamoto, Japan; 864 Department of Emergency and Critical Care Medicine, Tokyo Metropolitan Police Hospital, Tokyo, Japan; 865 Department of Respiratory Medicine, International University of Health and Welfare Shioya Hospital, Narita, Japan; 866 Department of Clinical Laboratory, International University of Health and Welfare Shioya Hospital, Narita, Japan; 867 National Hospital Organization Saitama Hospital, Saitama, Japan; 868 Department of Respiratory Medicine, Gunma University Graduate School of Medicine, Maebashi, Japan; 869 Department of Orthopedic Surgery, Tokyo Medical University, Ibaraki Medical Center, Tokyo, Japan; 870 Department of Internal Medicine, Kiryu Kosei General Hospital, Kiryu, Japan; 871 Daini Osaka Police Hospital, Osaka, Japan; 872 Genome Medical Science Project (Konodai), National Center for Global Health and Medicine, Chiba, Japan; 873 Ministry Of Health, Amman, Jordan; 874 School of Dentistry, The University of Jordan, Amman, Jordan; 875 School of Medicine, The University of Jordan, Amman, Jordan; 876 Cell Therapy Center, The University of Jordan, Amman, Jordan; 877 King Hussein Cancer Center, Amman, Jordan; 878 King Abdullah University Hospital, Amman, Jordan; 879 Gardens Hospital, Amman, Jordan; 880 Centre for Heart Lung Innovation, University of British Columbia, Vancouver, British Columbia Canada; 881 Division of Respiratory Medicine, Faculty of Medicine, University of British Columbia, Vancouver, British Columbia Canada; 882 Institut Universitaire de Cardiologie et de Pneumologie de Québec—Université Laval, Quebec City, Quebec Canada; 883 Department of Genetics and Genomic Sciences, Icahn School of Medicine at Mount Sinai, New York, NY USA; 884 Global Health, University of Washington, Seattle, WA USA; 885 Monoceros Bio, San Diego, CA USA; 886 Department of Pathology and Medical Biology Groningen, University Medical Centre Groningen, University of Groningen, Groningen, The Netherlands; 887 GRIAC Research Institute, University Medical Centre Groningen, University of Groningen, Groningen, The Netherlands; 888 Department of Pulmonary Diseases, University Medical Center Groningen, University of Groningen, Groningen, The Netherlands; 889 Center for Genomic Medicine, Massachusetts General Hospital, Boston, MA USA; 890 Institute of Epidemiology and Preventive Medicine, National Taiwan University, Taipei, Taiwan; 891 Master of Public Health Program, National Taiwan University, Taipei, Taiwan; 892 Channing Division of Network Medicine, Department of Medicine, Brigham and Women’s Hospital, Boston, MA USA; 893 Brigham and Women’s Hospital, Boston, MA USA; 894 Psychiatric and Neurodevelopmental Genetics Unit, Center for Genomic Medicine, Massachusetts General Hospital, Boston, MA USA; 895 Department of Neurology, Massachusetts General Hospital, Boston, MA USA; 896 Division of General Internal Medicine, Massachusetts General Hospital and Department of Medicine, Harvard Medical School and Program in Medical and Population GeneticsBroad Institute, Boston, MA USA; 897 Division of Genetics, Department of Medicine, Brigham and Women’s Hospital, Broad Institute of MIT and Harvard, Harvard Medical School, Boston, MA USA; 898 Department of Human Genetics, David Geffen School of Medicine at UCLA, Los Angeles, CA USA; 899 Departamento de Neurología y Psiquiatría, Instituto Nacional de Ciencias Médicas y Nutrición Salvador Zubirán, Tlalpan, Mexico City, Mexico; 900 Dirección de Medicina, Instituto Nacional de Ciencias Médicas y Nutrición Salvador Zubirán, Tlalpan, Mexico City, Mexico; 901 Infectología, Instituto Nacional de Ciencias Médicas y Nutrición Salvador Zubirán, Tlalpan, Mexico City, Mexico; 902 Clínica de Obesidad, Instituto Nacional de Ciencias Médicas y Nutrición Salvador Zubirán, Tlalpan, Mexico City, Mexico; 903 Unidad de Investigación en Enfermedades Metabólicas (UIEM), Instituto Nacional de Ciencias Médicas y Nutrición Salvador Zubirán, Tlalpan, Mexico City, Mexico; 904 Tecnologico de Monterrey, The Institute for Obesity Research, Nuevo León, Monterrey, Mexico; 905 Tecnologico de Monterrey, Escuela de Medicina y Ciencias de la Salud, Monterrey, Mexico; 906 Departamento de Endocrinología y Metabolismo de Lípidos, Instituto Nacional de Ciencias Médicas y Nutrición Salvador Zubirán, Tlalpan, Mexico City, Mexico; 907 Departamento de Psiquiatría y Salud Mental, Facultad de Medicina, Universidad Nacional Autónoma de México, Mexico City, Mexico; 908 Unidad de Biología Molecular y Medicina Genómica, Instituto Nacional de Ciencias Médicas y Nutrición Salvador Zubirán/Instituto de Investigaciones Biomédicas UNAM, Tlalpan, México; 909 Universidad Nacional Autónoma de México, Mexico City, Mexico; 910 Institute for Precision Health, David Geffen School of Medicine at UCLA, Los Angeles, CA USA; 911 Unidad de Biología Molecular y Medicina Genómica, Instituto Nacional de Ciencias Médicas y Nutrición Salvador Zubirán, CDMX, Tlalpan, México; 912 Universidad Autónoma Metropolitana, Mexico City, Mexico; 913 Departamento de Medicina Genómica y Toxicología Ambiental, Instituto de Investigaciones Biomédicas, UNAM, Mexico City, Mexico; 914 Department of Computer Science, School of Engineering, UCLA, Los Angeles, CA USA; 915 Unidad de Biología Molecular y Medicina Genómica, Instituto Nacional de Ciencias Médicas y Nutrición Salvador Zubirán, Tlalpan, Mexico City, Mexico; 916 Laboratorio de Microbiología, Instituto Nacional de Ciencias Médicas y Nutrición Salvador Zubirán, Tlalpan, Mexico City, Mexico; 917 Laboratorio de Neuropsicología y Cognición, Facultad de Psicología, Universidad Nacional Autónoma de México, Mexico City, Mexico; 918 Instituto Nacional de Ciencias Médicas y Nutrición Salvador Zubirán, Tlalpan, Mexico City, Mexico; 919 Facultad de Psicología, Universidad Nacional Autónoma de México, Mexico City, Mexico; 920 Facultad de Medicina, Universidad Nacional Autónoma de México, Mexico City, Mexico; 921 Laboratorio de Neuropsicología, Facultad de Medicina, Universidad Nacional Autónoma de México, UNAM, Coyoacán, Mexico City, Mexico; 922 Research Division, School of Medicine, Universidad Nacional Autónoma de México, Mexico City, Mexico; 923 Department of Computational Medicine, David Geffen School of Medicine at UCLA, Los Angeles, CA USA; 924 Department of Pathology and Laboratory Medicine, David Geffen School of Medicine at UCLA, Los Angeles, CA USA; 925 Division of Immunology, Allergy and Rheumatology, Department of Pediatrics, UCLA, Los Angeles, CA USA; 926 Department of Microbiology, Immunology, and Molecular Genetics, UCLA, Los Angeles, CA USA; 927 Department of Neurology, David Geffen School of Medicine at UCLA, Los Angeles, CA USA; 928 Department of Psychiatry and Biobehavioral Sciences, Semel Institute, David Geffen School of Medicine at UCLA, Los Angeles, CA USA; 929 Center for Autism Research and Treatment, Semel Institute, David Geffen School of Medicine at UCLA, Los Angeles, CA USA; 930 Institute for Precision Health, Los Angeles, CA USA; 931 Dirección de Nutrición, Instituto Nacional de Ciencias Médicas y Nutrición Salvador Zubirán, Tlalpan, Mexico City, Mexico; 932 Deutsches Herzzentrum der Charité, Universitätsmedizin, Berlin, Germany; 933 Charite Universitätsmedizin Berlin, Berlin, Germany; 934 Department of Tropical Medicine, Bernhard Nocht Institute for Tropical Medicine, Hamburg, Germany; 935 Department of Medicine I, University Medical Centre Hamburg-Eppendorf, Hamburg, Germany; 936 Qatar Genome Program, Qatar Foundation Research, Development and Innovation, Qatar Foundation, Doha, Qatar; 937 Directorate of Research, Shifa Tameer-e-Millat University, Islamabad, Pakistan; 938 Shifa Tameer-e-Millat University, Islamabad, Pakistan; 939 Department of Vascular Surgery, Shifa International Hospital, Islamabad, Pakistan; 940 Department of Infectious Diseases, Shifa International Hospital, Islamabad, Pakistan; 941 Shifa International Hospital, Islamabad, Pakistan; 942 Department of Pathology, Shifa International Hospital, Islamabad, Pakistan; 943 MNM Bioscience, Cambridge, MA USA; 944 Faculty of Physics, Adam Mickiewicz University, Poznań, Poland; 945 Department of Medical Chemistry and Laboratory Medicine, Poznan University of Medical Sciences, Poznan, Poland; 946 Central Clinical Hospital of Ministry of the Interior and Administration in Warsaw, Warsaw, Poland; 947 Institute of Genetics and Biotechnology, Faculty of Biology, University of Warsaw, Warsaw, Poland; 948 Genomics Division, Instituto Tecnológico y de Energías Renovables, Santa Cruz de Tenerife, Spain; 949 Research Unit, Hospital Universitario N.S. de Candelaria, Santa Cruz de Tenerife, Spain; 950 Centre for Biomedical Network Research on Respiratory Diseases (CIBERES), Instituto de Salud Carlos III, Madrid, Spain; 951 Facultad de Ciencias de la Salud, Universidad Fernando Pessoa Canarias, Las Palmas de Gran Canaria, Spain; 952 Centro Nacional de Genotipado (CEGEN), Universidade de Santiago de Compostela, Santiago de Compostela, Spain; 953 Centre for Biomedical Network Research on Rare Diseases (CIBERER), Instituto de Salud Carlos III, Madrid, Spain; 954 Fundación Pública Galega de Medicina Xenómica, Sistema Galego de Saúde (SERGAS) Santiago de Compostela, Santiago de Compostela, Spain; 955 Instituto de Investigación Sanitaria de Santiago (IDIS), Santiago de Compostela, Spain; 956 Centro Singular de Investigación en Medicina Molecular y Enfermedades Crónicas (CIMUS), Universidade de Santiago de Compostela, Santiago de Compostela, Spain; 957 Hospital General Santa Bárbara de Soria, Soria, Spain; 958 Biocruces Bizkai HRI, Bizkaia, Spain; 959 Cruces University Hospital, Osakidetza, Bizkaia, Spain; 960 Fundació Docència I Recerca Mutua Terrassa, Barcelona, Spain; 961 Hospital General de Occidente, Zapopan, Mexico; 962 Centro Universitario de Tonalá, Universidad de Guadalajara, Tonalá, Mexico; 963 Centro de Investigación Multidisciplinario en Salud, Universidad de Guadalajara, Tonalá, Mexico; 964 Laboratorio de Vigilancia Molecular Aplicada, Escola Tecnica de Saúde, Pará, Brazil; 965 Genetics Postgraduate Program, Federal University of Pernambuco, Recife, Brazil; 966 Cardiovascular Genetics Center, Institut d’Investigació Biomèdica Girona (IDIBGI), Girona, Spain; 967 Medical Science Department, School of Medicine, University of Girona, Girona, Spain; 968 Centre for Biomedical Network Research on Cardiovascular Diseases (CIBERCV), Instituto de Salud Carlos III, Madrid, Spain; 969 Cardiology Service, Hospital Josep Trueta, Girona, Spain; 970 Institute of Biomedicine of Seville (IBiS), Consejo Superior de Investigaciones Científicas (CSIC), University of Seville, Virgen del Rocio University Hospital, Seville, Spain; 971 Departamento de Medicina, Hospital Universitario Virgen del Rocío, Universidad de Sevilla, Seville, Spain; 972 Centre for Biomedical Network Research on Epidemiology and Public Health (CIBERESP), Instituto de Salud Carlos III, Madrid, Spain; 973 Instituto de Biomedicina de Sevilla, Seville, Spain; 974 Osakidetza, Cruces University Hospital, Bizkaia, Spain; 975 Centre for Biomedical Network Research on Diabetes and Metabolic Associated Diseases (CIBERDEM), Instituto de Salud Carlos III, Madrid, Spain; 976 University of Pais Vasco, UPV/EHU, Bizkaia, Spain; 977 Hospital Universitario Río Hortega, Valladolid, Spain; 978 Servicio de Medicina intensiva, Complejo Hospitalario Universitario de A Coruña (CHUAC), Sistema Galego de Saúde (SERGAS), A Coruña, Spain; 979 Instituto Aragonés de Ciencias de la Salud (IACS), Zaragoza, Spain; 980 Instituto Investigación Sanitaria Aragón (IIS-Aragon), Zaragoza, Spain; 981 Unidad Diagnóstico Molecular. Fundación Rioja Salud, La Rioja, Spain; 982 IDIVAL, Santander, Spain; 983 Universidad de Cantabria, Santander, Spain; 984 Hospital U M Valdecilla, Santander, Spain; 985 Hospital Universitario de Getafe, Servicio de Genética, Madrid, Spain; 986 IIS La Fe, Plataforma de Farmacogenética, Valencia, Spain; 987 Departamento de Farmacología, Universidad de Valencia, Valencia, Spain; 988 Facultad de Ciencias, Universidad de los Andes, Bogotá, Colombia; 989 SIGEN Alianza Universidad de los Andes—Fundación Santa Fe de Bogotá, Bogotá, Colombia; 990 Hospital General de Segovia, Medicina Intensiva, Segovia, Spain; 991 Instituto de Biomedicina (IBIOMED), Universidad de León, León, Spain; 992 Departamento Patologia y Laboratorios, Fundación Santa Fe de Bogota, Bogotá, Colombia; 993 Unidad de Genética y Genómica Islas Baleares, Islas Baleares, Spain; 994 Unidad de Diagnóstico Molecular y Genética Clínica, Hospital Universitario Son Espases, Islas Baleares, Spain; 995 Faculdade de Medicina, Universidade de Brasília, Brasília, Brazil; 996 Programa de Pós-Graduação em Ciências Médicas, Universidade de Brasília, Brasília, Brazil; 997 Programa de Pós-Graduação em Ciências da Saúde, Universidade de Brasília, Brasília, Brazil; 998 Unidad Cuidados Intensivos, Hospital El Bierzo, León, Spain; 999 Medicina Interna, Hospital Universitario Mostoles, Madrid, Spain; 1000 Universidad Francisco de Vitoria, Madrid, Spain; 1001 Departamento de Analises Clinicas e Toxicologicas, Universidade Federal do Rio Grande do Norte, Natal, Brazil; 1002 Programa de Pós-Graduação em Biologia Animal, Universidade de Brasília, Brasília, Brazil; 1003 Programa de Pós-Graduação Profissional em Ensino de Biologia, Universidade de Brasília, Brasília, Brazil; 1004 Department of Child and Adolescent Psychiatry, Institute of Psychiatry and Mental Health, Hospital General Universitario Gregorio Marañón (IiSGM), Madrid, Spain; 1005 Centre for Biomedical Network Research on Mental Health (CIBERSAM), Instituto de Salud Carlos III, Madrid, Spain; 1006 School of Medicine, Universidad Complutense, Madrid, Spain; 1007 Núcleo de Pesquisas em Oncologia, Universidade Federal do Pará, Belém, Brazil; 1008 Infectious Diseases, Microbiota and Metabolism Unit, Center for Biomedical Research of La Rioja (CIBIR), Logroño, Spain; 1009 Inditex, A Coruña, Spain; 1010 GENYCA, Madrid, Spain; 1011 Clinica Comfamiliar Risaralda, Pereira, Colombia; 1012 Neurometabolic Diseases Laboratory, Bellvitge Biomedical Research Institute (IDIBELL), L’Hospitalet de Llobregat, Spain; 1013 Catalan Institution of Research and Advanced Studies (ICREA), Barcelona, Spain; 1014 Unidad de Infección Viral e Inmunidad, Centro Nacional de Microbiología (CNM), Instituto de Salud Carlos III (ISCIII), Madrid, Spain; 1015 Centro de Investigación Biomédica en Red de Enfermedades Infecciosas (CIBERINFEC), Instituto de Salud Carlos III, Madrid, Spain; 1016 Unidad de Cuidados Intensivos, Hospital Clínico Universitario de Santiago (CHUS), Sistema Galego de Saúde (SERGAS), Santiago de Compostela, Spain; 1017 Department of Preventive Medicine and Public Health, School of Medicine, Universidad Autónoma de Madrid, Madrid, Spain; 1018 IdiPaz (Instituto de Investigación Sanitaria Hospital Universitario La Paz), Madrid, Spain; 1019 IMDEA-Food Institute, CEI UAM+CSIC, Madrid, Spain; 1020 Complejo Asistencial Universitario de León, León, Spain; 1021 Servicio de Análisis Clínicos e Inmunología, Hospital Universitario Virgen de las Nieves, Granada, Spain; 1022 Departamento Bioquímica, Biología Molecular e Inmunología III, Universidad de Granada, Granada, Spain; 1023 Genomics of Complex Diseases Unit, Research Institute of Hospital de la Santa Creu i Sant Pau, IIB Sant Pau, Barcelona, Spain; 1024 Servicio de Anestesiologia y Reanimación, Hospital Clinico Universitario de Valladolid, Valladolid, Spain; 1025 Departamento de Cirugía, Universidad de Valladolid, Valladolid, Spain; 1026 Hospital de Niños Ricardo Gutierrez, Buenos Aires, Argentina; 1027 Fundación Universitaria de Ciencias de la Salud, Bogotá, Colombia; 1028 Familial Cancer Clinical Unit, Spanish National Cancer Research Centre, Madrid, Spain; 1029 Life Science Research Center, Universidad Simón Bolívar, Barranquilla, Colombia; 1030 Instituto Murciano de Investigación Biosanitaria (IMIB-Arrixaca), Murcia, Spain; 1031 Sección Genética Médica—Servicio de Pediatría, Hospital Clínico Universitario Virgen de la Arrixaca, Servicio Murciano de Salud, Murcia, Spain; 1032 Departamento Cirugía, Pediatría, Obstetricia y Ginecología, Facultad de Medicina, Universidad de Murcia (UMU), Murcia, Spain; 1033 Grupo Clínico Vinculado, Centre for Biomedical Network Research on Rare Diseases (CIBERER), Instituto de Salud Carlos III, Madrid, Spain; 1034 Department of Genetics & Genomics, Instituto de Investigación Sanitaria-Fundación Jiménez Díaz University Hospital—Universidad Autónoma de Madrid (IIS-FJD, UAM), Madrid, Spain; 1035 Human Genotyping-CEGEN Unit, Spanish National Cancer Research Centre, Madrid, Spain; 1036 Instituto de Genética Médica y Molecular (INGEMM), Hospital Universitario La Paz-IDIPAZ, Madrid, Spain; 1037 ERN-ITHACA—European Reference Network, Madrid, Spain; 1038 Unit of Infectious Diseases, Hospital Universitario 12 de Octubre, Instituto de Investigación Sanitaria Hospital 12 de Octubre (imas12), Madrid, Spain; 1039 Spanish Network for Research in Infectious Diseases (REIPI RD16/0016/0002), Instituto de Salud Carlos III, Madrid, Spain; 1040 Pediatric Neurology Unit, Department of Pediatrics, Navarra Health Service Hospital, Pamplona, Spain; 1041 Navarra Health Service, NavarraBioMed Research Group, Pamplona, Spain; 1042 Neumología, Hospital Universitario Virgen Macarena, Seville, Spain; 1043 Spanish National Cancer Research Center, CNIO Biobank, Madrid, Spain; 1044 Universidad Católica San Antonio de Murcia (UCAM), Murcia, Spain; 1045 Servicio de Medicina Interna-Unidad de Enfermedades Infecciosas, Hospital Universitario de Salamanca-IBSAL, Salamanca, Spain; 1046 Universidad de Salamanca, Salamanca, Spain; 1047 Hospital Universitario Mutua Terrassa, Barcelona, Spain; 1048 Xenética Cardiovascular, Instituto de Investigación Sanitaria de Santiago (IDIS), Santiago de Compostela, Spain; 1049 Servicio de Medicina Interna, Hospital Universitario de Salamanca-IBSAL, Salamanca, Spain; 1050 Oncology and Genetics Unit, Instituto de Investigacion Sanitaria Galicia Sur, Xerencia de Xestion Integrada de Vigo-Servizo Galego de Saúde, Vigo, Spain; 1051 Departament of Microgravity and Translational Regenerative Medicine, Otto von Guericke University, Magdeburg, Germany; 1052 Unidad de Genética, Hospital Universitario Mostoles, Madrid, Spain; 1053 Preventive Medicine Department, Instituto de Investigacion Sanitaria Galicia Sur, Xerencia de Xestion Integrada de Vigo-Servizo Galego de Saúde, Vigo, Spain; 1054 Servicio de Cardiología, Hospital Universitario de Salamanca-IBSAL, Salamanca, Spain; 1055 Instituto Regional de Investigación en Salud-Universidad Nacional de Caaguazú, Caaguazú, Paraguay; 1056 Urgencias Hospitalarias, Complejo Hospitalario Universitario de A Coruña (CHUAC), Sistema Galego de Saúde (SERGAS), A Coruña, Spain; 1057 Grupo de Investigación en Interacciones Gen-Ambiente y Salud (GIIGAS), Instituto de Biomedicina (IBIOMED), Universidad de León, León, Spain; 1058 Ministerio de Salud Ciudad de Buenos Aires, Buenos Aires, Argentina; 1059 Data Analysis Department, Instituto de Investigación Sanitaria-Fundación Jiménez Díaz University Hospital, Universidad Autónoma de Madrid (IIS-FJD, UAM), Madrid, Spain; 1060 Department of Immunology, Hospital Universitario 12 de Octubre, Madrid, Spain; 1061 Transplant Immunology and Immunodeficiencies Group, Instituto de Investigación Sanitaria Hospital 12 de Octubre (imas12), Madrid, Spain; 1062 Department of Immunology, Ophthalmology and ENT, Universidad Complutense de Madrid, Madrid, Spain; 1063 Unidad de Investigación Médica en Enfermedades Infecciosas y Parasitarias, Centro Médico Nacional Siglo XXI, Instituto Mexicano del Seguro Social (IMSS), Mexico City, Mexico; 1064 Centro de Investigación Biomédica en Red de Enfermedades Infecciosas (CIBERINFEC), Instituto de Salud Carlos III (ISCIII), Madrid, Spain; 1065 Departamento de Ensino e Pesquisa, Hospital Ophir Loyola, Belém, Brazil; 1066 Allergy Unit, Hospital Infanta Elena, Madrid, Spain; 1067 Instituto de Investigación Sanitaria-Fundación Jiménez Díaz University Hospital—Universidad Autónoma de Madrid (IIS-FJD, UAM), Madrid, Spain; 1068 Faculty of Medicine, Universidad Francisco de Vitoria, Madrid, Spain; 1069 Hospital Universitario Infanta Leonor, Madrid, Spain; 1070 Complutense University of Madrid, Madrid, Spain; 1071 Gregorio Marañón Health Research Institute (IiSGM), Madrid, Spain; 1072 Haemostasis and Thrombosis Unit, Hospital de la Santa Creu i Sant Pau, IIB Sant Pau, Barcelona, Spain; 1073 Servicio de Hematologia y Hemoterapia, Hospital Clinico Universitario de Valladolid, Valladolid, Spain; 1074 University Hospital of Burgos, Burgos, Spain; 1075 Department of Clinical Immunology, Aarhus University Hospital, Aarhus, Denmark; 1076 Department of Clinical Immunology, Copenhagen University Hospital—Rigshospitalet, Copenhagen, Denmark; 1077 deCODE genetics, Reykjavik, Iceland; 1078 Department of Finance Copenhagen Business School, Copenhagen, Denmark; 1079 Danish Headache Center, Department of Neurology, Copenhagen University Hospital, Rigshospitalet—Glostrup, Copenhagen, Denmark; 1080 Centre for Cancer Research, Danish Cancer Society, Copenhagen, Denmark; 1081 Department of Dermatology, Zealand University hospital, Roskilde, Denmark; 1082 Department of Biomedicine, Aarhus University, Aarhus, Denmark; 1083 Institute of Biological Psychiatry, Mental Health Centre Sct. Hans, Copenhagen University Hospital, Roskilde, Denmark; 1084 deCODE Genetics, Reykjavik, Iceland; 1085 Department of Clinical Immunology, Aalborg University Hospital, Aalborg, Denmark; 1086 Department of Clinical Immunology, Odense University Hospital, Odense, Denmark; 1087 Department of Neuroscience, Karolinska Institutet, Stockholm, Sweden; 1088 Max Planck Institute for Evolutionary Anthropology, Leipzig, Germany; 1089 Stanley Center for Psychiatric Research & Program in Medical and Population Genetics, Cambridge, UK; 1090 Anaesthesiology and Intensive Care Medicine, Department of Surgical Sciences, Uppsala University, Uppsala, Sweden; 1091 Integrative Physiology, Department of Medical Cell Biology, Uppsala University, Uppsala, Sweden; 1092 Hedenstierna Laboratory, CIRRUS, Anaesthesiology and Intensive Care Medicine, Department of Surgical Sciences, Uppsala University, Uppsala, Sweden; 1093 Division Anesthesiology and Intensive Care, CLINTEC, Karolinska Institutet, Stockholm, Sweden; 1094 Department of Genetic Epidemiology, University of Regensburg, Regensburg, Germany; 1095 Institute of Medical Microbiology and Hygiene, Molecular Microbiology (Virology), University of Regensburg, Regensburg, Germany; 1096 Virologisches Institut—Klinische und Molekulare Virologie, Universitätsklinikum Erlangen, Friedrich-Alexander Universität Erlangen-Nürnberg (FAU), Erlangen, Germany; 1097 Institute of Clinical Microbiology and Hygiene, University Hospital, Regensburg, Germany; 1098 Department of Computer Science, School of Engineering, University of California Los Angeles, Los Angeles, CA USA; 1099 University of California Los Angeles, Los Angeles, CA USA; 1100 Department of Psychiatry and Biobehavioral Sciences, David Geffen School of Medicine at University of California Los Angeles, Los Angeles, CA USA; 1101 Division of Immunology, Allergy and Rheumatology, Department of Pediatrics, Department of Microbiology, Immunology and Molecular Genetics, University of California Los Angeles, Los Angeles, CA USA; 1102 Departments of Neurology, Psychiatry and Human Genetics, Center for Autism Research and Treatment, Institute for Precision Health, Los Angeles, CA USA; 1103 Department of Pathology and Laboratory Medicine, Human Genetics, David Geffen School of Medicine at UCLA, Los Angeles, CA USA; 1104 Bioinformatics IDP, University of California Los Angeles, Los Angeles, CA USA; 1105 Department of Neurology, David Geffen School of Medicine at University of California Los Angeles, Los Angeles, CA USA; 1106 Departments of Human Genetics and Urology, David Geffen School of Medicine at University of California Los Angeles, Los Angeles, CA USA; 1107 Big Data Institute, Nuffield Department of Population Health, University of Oxford, Li Ka Shing Centre for Health Information and Discovery, Oxford, UK; 1108 Genomics PLC, Oxford, UK; 1109 Nuffield Department of Medicine, Experimental Medicine Division, University of Oxford, John Radcliffe Hospital, Oxford, UK; 1110 Public Health England, Field Service, Addenbrooke’s Hospital, Cambridge, UK; 1111 Public Health England, Data and Analytical Services, National Infection Service, London, UK; 1112 Department of Molecular and Human Genetics, Baylor College of Medicine, Houston, TX USA; 1113 Program in Bioinformatics and Integrative Genomics, Harvard Medical School, Boston, MA USA; 1114 Program in Biological and Biomedical Sciences, Harvard Medical School, Boston, MA USA; 1115 Center for Economic and Social Research, University of Southern California, Los Angeles, CA USA; 1116 Department of Economics, University of Southern California, Los Angeles, CA USA; 1117 Institute for Molecular Medicine Finland (FIMM), Univerisity of Helsinki, Helsinki, Finland; 1118 Wellcome Centre for Human Genetics, University of Oxford, Oxford, UK; 1119 Department of Clinical Research and Leadership, George Washington University, Washington, DC USA; 1120 Institute for Biomedical Research of Barcelona (IIBB), National Spanish Research Council (CSIC), Madrid, Spain; 1121 Institut d’Investigacions Biomèdiques August Pi i Sunyer (IDIBAPS), Barcelona, Spain; 1122 Institute of Biomedicine of Valencia (IBV), CSIC, Valencia, Spain; 1123 Network Center for Biomedical Research on Neurodegenerative Diseases CIBERNED, Instituto de Salud Carlos III (ISCIII), Valencia, Spain; 1124 Institute for Biomedical Research of Barcelona (IIBB), National Spanish Research Council (CSIC), Madrid, Spain; 1125 Department of Neurology, Hospital Universitari MútuaTerrassa, Fundació Docència i Recerca MútuaTerrassa, Terrassa, Spain; 1126 Department of Molecular and Cell Biology, Centro Nacional de Biotecnología (CNB-CSIC), Campus Universidad Autónoma de Madrid, Madrid, Spain; 1127 Instituto de Física de Cantabria (IFCA-CSIC), Santander, Spain; 1128 Hospital Clínic, IDIBAPS, Barcelona, Spain; 1129 Hospital Clínic, Barcelona, Spain; 1130 Hospital Clínic, IDIBAPS, School of Medicine, University of Barcelona Barcelona, Barcelona, Spain; 1131 IDIBAPS, Barcelona, Spain; 1132 IIBB-CSIC, Barcelona, Spain; 1133 Servicio de Salud del Principado de Asturias, Oviedo, Spain; 1134 Hospital Mutua de Terrassa, Terrassa, Spain; 1135 Vall d’Hebron Research Institute, Vall d’Hebron Barcelona Hospital Valle Hebrón Campus, Barcelona, Spain; 1136 Unidad de Excelencia Instituto de Biomedicina y Genética Molecular (IBGM, Universidad de Valladolid-CSIC), Valladolid, Spain; 1137 Hospital Clínico Universitario de Valladolid (SACYL), Valladolid, Spain; 1138 Department of Neurology, University Hospital of Albacete, Albacete, Spain; 1139 Research Unit, University Hospital of Albacete, Albacete, Spain; 1140 Department of Neurology, Biomedical Research Institute Sant Pau (IIB Sant Pau), Hospital de la Santa Creu i Sant Pau, Barcelona, Spain; 1141 Hospital Universitario Ramón Y Cajal, IRYCIS, Madrid, Spain; 1142 Institute de Biomedicine of Seville, IBiS/Hospital Universitario Virgen del Rocío/CSIC/University of Seville & Department of Neurology, Hospital Universitario Virgen Macarena, Seville, Spain

**Keywords:** Genome-wide association studies, SARS-CoV-2

arising from: COVID-19 Host Genetics Initiative. *Nature* 10.1038/s41586-021-03767-x (2021)

Investigating the role of host genetic factors in COVID-19 severity and susceptibility can inform our understanding of the underlying biological mechanisms that influence adverse outcomes and drug development^1,2^. Here we present a second updated genome-wide association study (GWAS) on COVID-19 severity and infection susceptibility to SARS-CoV-2 from the COVID-19 Host Genetic Initiative (data release 7). We performed a meta-analysis of up to 219,692 cases and over 3 million controls, identifying 51 distinct genome-wide significant loci—adding 28 loci from the previous data release^2^. The increased number of candidate genes at the identified loci helped to map three major biological pathways that are involved in susceptibility and severity: viral entry, airway defence in mucus and type I interferon.

We conducted a meta-analysis for 3 phenotypes across 82 studies from 35 countries, including 36 studies of individuals with non-European ancestry (Fig. 1, Supplementary Figs. 1 and 2 and Supplementary Table 1): critical illness (respiratory support or death; 21,194 cases), hospitalization (49,033 cases) and SARS-CoV-2 infection (219,692 cases). Most of the studies were collected before the widespread introduction of COVID-19 vaccination. We found 30, 40 and 21 loci that are associated with critical illness, hospitalization and infection due to SARS-CoV-2, respectively, for a total of 51 distinct genome-wide significant loci across all three phenotypes (*P* < 5 × 10^−8^; Fig. 2, Supplementary Fig. 3 and Supplementary Table 2), adding 28 genome-wide significant loci to the 23 previously identified by the COVID-19 Host Genomics Initiative (HGI; data release 6)^1,2^. We observed a median increase of 2.9-fold in statistical power across lead variants owing to a median increase of 1.6-fold in effective sample sizes from the previous release (Supplementary Table 3). After correcting for the number of phenotypes examined, 46 loci remained significant (*P* < 1.67 × 10^−8^). Of the 28 additional loci, 6 loci were originally reported by the GenOMICC study^3^, which also contributed to the current meta-analysis, and 9 other loci were identified by the new GenOMICC meta-analysis^4^ during the preparation of this paper. We found nine more loci that reached genome-wide significance, but we excluded them as they were probably false positives, as determined using a leave-most-significant-biobank-out analysis (Supplementary Table 4 and Supplementary Note). Comparing the effect sizes and statistical significance between the previous^2^ and current analysis indicated that all of the previously identified loci were replicated and showed an increase in statistical significance (Supplementary Fig. 4). Using our previously developed two-class Bayesian model for classifying loci as being more likely involved in infection susceptibility or severity^2^, we determined that 36 loci are substantially more likely (higher than 99% posterior probability) to affect disease severity (hospitalization) and 9 loci are substantially more likely to influence susceptibility to SARS-CoV-2 infection, while the remaining 6 loci could not be classified (Supplementary Fig. 5, Supplementary Table 5 and Supplementary Note). We observed that the 1q22 locus (lead variant: rs12752585:G>A) showed significant effect-size heterogeneity across ancestries (*P*_het_ < 9.80 × 10^−4^ = 0.05/51), whereas the previously reported heterogenous locus (*FOXP4*) remained at the same level of significance as before^2^, despite an increase in sample size (*P*_het_ = 2.01 × 10^−3^; Supplementary Fig. 6 and Supplementary Table 6). We found significant observed-scale single-nucleotide polymorphism heritabilities of all the three phenotypes (1.2–8.2%, *P* < 0.0001). We also estimated liability-scale heritabilities for a range of population prevalences (Supplementary Fig. 7, Supplementary Table 7 and Supplementary Note).

**Fig. 1:**
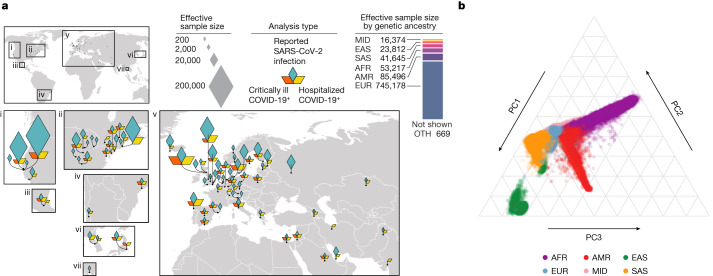
Overview of the contributing studies in the HGI data release 7. **a**, Geographical overview of the studies contributing to the COVID-19 HGI and the composition by major ancestry groups. Populations are defined as Middle Eastern (MID), South Asian (SAS), East Asian (EAS), African (AFR), admixed American (AMR), European (EUR) and other (OTH). **b**, A principal component analysis (PCA) highlights the population structure and the sample ancestry of the individuals participating in the COVID-19 HGI. Per-cohort PCA results are provided in Supplementary Fig. 2. This figure was reproduced from the original publication by the COVID-19 HGI^1^ with modifications reflecting the updated analysis from data release 7.

**Fig. 2:**
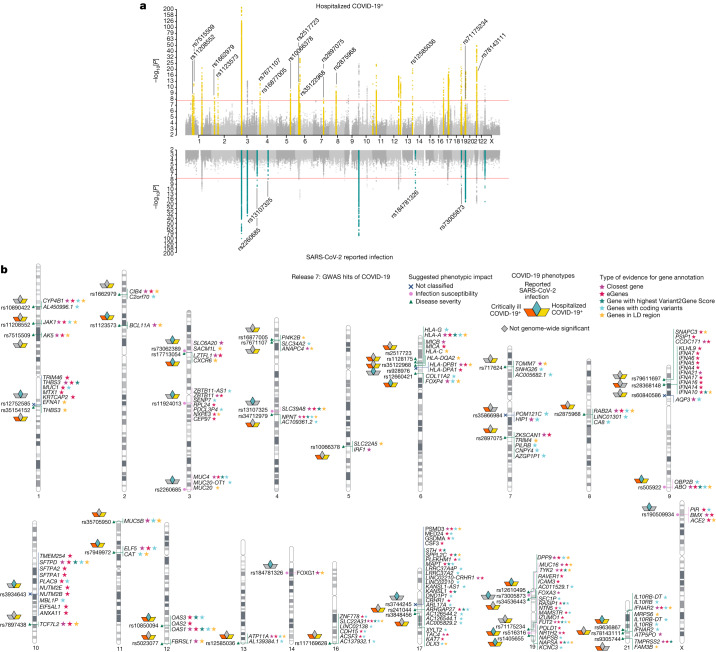
GWAS results for COVID-19. **a**, The results of a GWAS analysis of hospitalized individuals with COVID-19 (*n* = 49,033 cases and *n* = 3,393,109 controls) (top), and the results for individuals with reported SARS-CoV-2 infection (*n* = 219,692 cases and *n* = 3,001,905 controls) (bottom). The loci highlighted in yellow (top) represent regions that are associated with severity of COVID-19. The loci highlighted in green (bottom) are regions associated with susceptibility to SARS-CoV-2 infection. Lead variants for the loci that were identified in this data release are annotated with their respective rsID. The *y* axis is on the −log_10_ (*P*) scale up to 10, after which it switches to a 10 × log_10_[−log_10_(*P*)] scale to aid presentation. **b**, Results of gene prioritization using different evidence measures of gene annotation. For the genes in a linkage-disequilibrium (LD) region, genes with coding variants and eGenes (fine-mapped *cis*-expression quantitative trait locus (*cis*-eQTL) variant with posterior inclusion probability (PIP) >0.1 in GTEx Lung) are annotated as such if they are in linkage disequilibrium with a COVID-19 lead variant (*r*^2^ > 0.6). V2G, the highest gene prioritized by OpenTargetGenetics V2G score. The pink circle indicates SARS-CoV-2 infection susceptibility, the green triangle indicates COVID-19 severity and the blue cross indicates unclassified. This figure was reproduced from the original publication by the COVID-19 HGI^1^ with modifications reflecting the updated analysis from data release 7.

To better understand the biological mechanisms underlying COVID-19 susceptibility and severity, we further characterized candidate causal genes by mapping them onto biological pathways and performing a phenome-wide association analysis (Extended Data Fig. 1, Supplementary Fig. 8 and Supplementary Tables 2, 8 and 9). In total, 15 out of 51 loci could be linked to three major pathways involved in susceptibility and severity defined by expert-driven classification (Supplementary Note): (1) viral entry; (2) entry defence in airway mucus; and (3) type I interferon response. Moreover, the phenome-wide association analysis identified nine loci involved in the upkeep of healthy lung tissue.

First, five loci include candidate causal genes involved in the viral entry pathway (Extended Data Fig. 1a), such as previously reported *SLC6A20* (3p21.31), *ABO* (9q34.2), *SFTPD* (10q22.3) and *ACE2* (Xp22.2), as well as *TMPRSS2* (21q22.3), which was also identified in the data release 7. We found that the lead variant rs9305744:G>A, an intronic variant of *TMPRSS2*, is protective against critical illness (odds ratio (OR) = 0.92, 95% CI = 0.89–0.95, *P* = 1.4 × 10^−8^) and is in linkage disequilibrium with the missense variant rs12329760:C>T (p.Val197Met; *r*^2^ = 0.68). SARS-CoV-2 uses the serine protease TMPRSS2 for viral spike protein priming, as well as the previously reported ACE2 for host cell entry which functionally interacts with SLC6A20 (refs. ^5,6^). Notably, the previously reported association between ABO blood groups and susceptibility could be attributed to the interference of anti-A and anti-B antibodies with the spike protein, potentially interfering with viral entry^7^. Furthermore, the previously reported *SFTPD* encodes pulmonary surfactant protein D (SP-D), which contributes dually to the lung’s innate immune molecules and viral-entry response in pulmonary epithelia^8,9^ along with other genes for airway defence.

Second, four loci contain candidate causal genes for entry defence in the airway mucus (Extended Data Fig. 1b), such as previously reported *MUC1*/*THBS3* (1q22) and *MUC5B* (11p15.5) as well as novel *MUC4* (3q29) and *MUC16* (19p13.2). We found that the novel lead variants rs2260685:T>C in *MUC4* (intronic variant; in linkage disequilibrium (*r*^2^ = 0.65) with a missense variant rs2259292:C>T (p.Gly4324Asp)) and rs73005873:G>A in *MUC16* (intronic variant) increase the risk of SARS-CoV-2 infection (OR = 1.03 and 1.03, 95% CI = 1.02–1.04 and 1.02–1.04, *P* = 4.1 × 10^−^^8^ and 6.4 × 10^−10^, respectively). Moreover, the previously reported locus 1q22 contains an intergenic lead variant rs12752585:G>A that decreases the risk of infection (OR = 0.98, 95% CI = 0.97–0.98, *P* = 1.5 × 10^−^^11^) and increases *MUC1* expression in the oesophagus mucosa in GTEx v8 (*P* = 5.2 × 10^−9^). Notably, the 1q22 locus also contains an independent lead variant, rs35154152:T>C, a missense variant (p.Ser279Gly) of *THBS3*, that decreases the risk of hospitalization (OR = 0.88, 95% CI = 0.86–0.90, *P* = 5.6 × 10^−^^22^) but not infection (*P* = 5.7 × 10^−^^4^), suggesting potential distinct mechanisms in the locus. Consistent with these association patterns, MUC1, MUC4 and MUC16 are three known major transmembrane mucins of the respiratory tracts that prevent microbial invasion, whereas previously reported MUC5B, together with nearby MUC5AC, are primary structural components of airways mucus that enable mucociliary clearance of pathogens^10^.

Third, six loci contain candidate causal genes that are linked to the type I interferon pathway (Extended Data Fig. 1c), such as previously reported *IFNAR2* (21q22.11), *OAS1* (12q24.13) and *TYK2* (19p13.2), as well as additionally identified *JAK1* (1p31.3), *IRF1* (5q31.1) and IFNα-coding genes (9p21.3). Previous studies have reported additional genes in this pathway: *TLR7* (refs. ^11,12^) and *DOCK2* (ref. ^13^). Here we found that the lead variant rs28368148:C>G, a missense variant (p.Trp164Cys) of *IFNA10* located within the IFNα gene cluster, increases the risk of critical illness (OR = 1.56, 95% CI = 1.38–1.77, *P* = 3.7 × 10^−12^). IFNα is one of the type I interferons that binds specifically to the IFNα receptor consisting of IFNAR1–IFNAR2 chains, in which mutations are also known to increase the risk of hospitalization and critical illness. In the genes that enable signalling downstream of IFNAR, we identified that the lead variant rs11208552:G>T, an intronic variant of *JAK1*, is protective against critical illness and hospitalization (OR = 0.92 and 0.95, 95% CI = 0.89–0.94 and 0.93–0.96, *P* = 5.5 × 10^−10^ and 2.2 × 10^−^^9^, respectively). This variant was previously reported to decrease lymphocyte counts^14^ (*β* = −0.016, *P* = 5.5 × 10^−15^) and increase the *JAK1* expression in the thyroid in GTEx^15^ (*P* = 6.1 × 10^−23^). JAK1 and previously reported TYK2 are Janus kinases (JAKs) that are required for type I interferon-induced JAK–STAT signalling. JAK inhibitors are used to treat patients with severe COVID-19 (ref. ^16^). Furthermore, downstream of JAK–STAT signalling, we found that the lead variant rs10066378:T>C, located 67 kb upstream of *IRF1*, increases the risk of critical illness and hospitalization (OR = 1.09 and 1.07, 95% CI = 1.06–1.13 and 1.05–1.09, *P* = 2.7 × 10^−9^ and 3.74 × 10^−10^, respectively).

Furthermore, the phenome-wide association analysis identified nine loci previously associated with lung function and respiratory diseases. These loci contain genes involved in the upkeep of healthy lung tissue such as previously reported *FOXP4* (6p21.1), *SFTPD* (10q22.3), *MUC5B* (11p15.5) and *DPP9* (19p13.3), as well as additionally identified *CIB4* (2p23.3), *NPNT* (4q24), *ZKSCAN1* (7q22.1), *ATP11A* (13q34) and *PSMD3* (17q21.1). For example, we found that three lead variants, rs1662979:G>T (intronic variant of *CIB4*), rs34712979:G>A (splice region variant of *NPNT*) and rs2897075:C>T (intronic variant of *ZKSCAN1*), are significantly associated with hospitalization (OR = 1.05, 0.94 and 1.05, 95% CI = 1.03–1.07, 0.92–0.96 and 1.03–1.07, *P* = 5.6 × 10^−9^, 3.8 × 10^−8^ and 8.9 × 10^−9^, respectively) and lung function (FEV1/FVC)^17^, similar to the previously reported lead variant rs3934643:G>A (intronic variant of *SFTPD*). Notably, whereas the alleles associated with increased risk of COVID-19 severity of rs1662979 and rs3934643 decrease lung function (*β* = −0.013 and −0.025, *P* = 5.3 × 10^−8^ and 6.3 × 10^−10^), those of rs34712979 and rs2897075 increase lung function (*β* = 0.068 and 0.023, *P* = 4.2 × 10^−134^ and 1.6 × 10^−20^, respectively). Likewise, we found lead variants that were significantly associated with hospitalization and idiopathic pulmonary fibrosis^18,19^, such as the aforementioned rs2897075 and rs12585036:C>T (intronic variant of *ATP11A*; OR = 1.10, 95% CI = 1.08–1.12, *P* = 3.2 × 10^−21^), in addition to the previously reported rs35705950:G>T (promoter variant of *MUC5B*). Whereas the COVID-19 severity risk-increasing alleles of rs2897075 and rs12585036 increase the risk of idiopathic pulmonary fibrosis (OR = 1.12 and 1.27, *P* = 3.0 × 10^−14^ and 7.0 × 10^−9^, respectively), those of rs35705950 decreases the risk (OR = 0.50, *P* = 3.9 × 10^−80^). These results highlight the complex pleiotropic relationships between COVID-19 severity, lung function and respiratory diseases.

We used genetic correlations and Mendelian randomization analyses to identify potential causal effects of risk factors on COVID-19 phenotypes (Supplementary Fig. 9 and Supplementary Tables 10 and 11). In total, 14 novel genetic correlations and 10 novel robust exposure-COVID-19 trait pairs showed evidence of causal associations (Supplementary Note). In particular, smoking initiation and the number of cigarettes per day were positively correlated with severity and susceptibility phenotypes; Mendelian randomization indicated that smoking was causally associated with increased risk of COVID-19, further highlighting the role of the healthy lung tissue in COVID-19 severity. Moreover, genetically instrumented higher glomerular filtration rate (indicative of better kidney function) was associated with a lower risk of COVID-19 critical illness, whereas genetically predicted chronic kidney disease was associated with an increased risk of COVID-19 critical illness, suggesting that better kidney function would be beneficial for a lower risk of COVID-19 severity.

In summary, we have substantially expanded the current knowledge of host genetics for COVID-19 susceptibility and severity by further doubling the case numbers from the previous data release^2^ and identifying 28 additional loci. The increased number of loci enables us to map genes to pathways that are involved in viral entry, airway defence and immune system response. Notably, we observed severity loci mapped to type I interferon pathway, while susceptibility loci mapped to viral entry and airway defence pathways, with notable exceptions for severity-classified *TMPRSS2* and *MUC5B* loci. Further investigation of how such susceptibility and severity loci map to different pathways would provide mechanistic insights into the human genetic architecture of COVID-19.

## Reporting summary

Further information on research design is available in the Nature Portfolio Reporting Summary linked to this article.

## Online content

Any methods, additional references, Nature Portfolio reporting summaries, source data, extended data, supplementary information, acknowledgements, peer review information; details of author contributions and competing interests; and statements of data and code availability are available at 10.1038/s41586-023-06355-3.

## Supplementary information

Supplementary Information Supplementary Methods, Supplementary References, Supplementary Figs. 1–7 and 9, and the legend for Supplementary Fig. 8.

Reporting Summary

Supplementary Fig. 8 Supplementary Fig. 8.

Supplementary Tables Supplementary Tables 1–12.

## Figures and Tables

**Extended Data Fig. 1:**
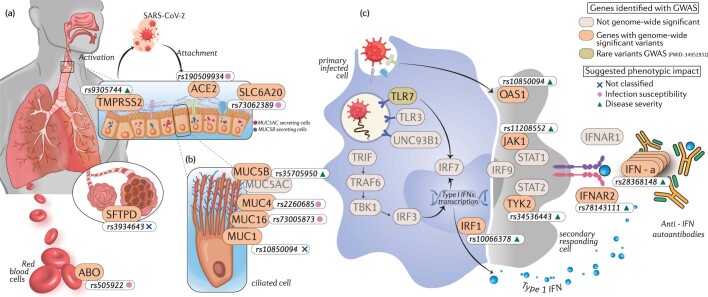
Major COVID-19 biological pathways mapped with susceptibility and severity GWAS loci. Genome-wide significant variants associated with COVID-19 (white boxes) and the annotated genes (peach boxes) are mapped on to pathways known to be involved in (**a**) viral entry and innate immunity, (**b**) entry defence in airway mucus, and (**c**) type I interferon. The suggested phenotypic impact of the significant variants using the Bayesian approach^2^ are denoted with shapes; COVID-19 susceptibility (pink circles), COVID-19 disease severity (green triangles), and unclassified variants (blue cross). Other genes known to be involved in the aforementioned pathways are shown using grey boxes. Detailed list of references to studies used to design this pathway can be found in Supplementary Note.

## Data Availability

Summary statistics generated by the COVID-19 HGI are available online, including per-ancestry summary statistics for African, admixed American, East Asian, European and South Asian ancestries (https://www.covid19hg.org/results/r7/). The analyses described here used the data release 7. If available, individual-level data can be requested directly from contributing studies, listed in Supplementary Table 1. We used publicly available data from GTEx (https://gtexportal.org/home/), the Neale laboratory (http://www.nealelab.is/uk-biobank/), the Finucane laboratory (https://www.finucanelab.org), the FinnGen Freeze 4 cohort (https://www.finngen.fi/en/access_results) and the eQTL catalogue release 3 (http://www.ebi.ac.uk/eqtl/).

## Code availability

The code for summary statistics lift-over, the projection PCA pipeline including precomputed loadings and meta-analyses (https://github.com/covid19-hg/); for heritability estimation (https://github.com/AndrewsLabUCSF/COVID19_heritability); for Mendelian randomization and genetic correlation (https://github.com/marcoralab/MRcovid); and subtype analyses (https://github.com/mjpirinen/covid19-hgi_subtypes) are available at GitHub.
