# Supplementary material for: A second update on mapping the human genetic architecture of COVID-19
Source: Nature. 2023 Sep 6;621(7977):E7–E26. doi: 10.1038/s41586-023-06355-3 (PMC10482689; doi:10.1038/s41586-023-06355-3)
Supplement: Supplementary file 2 — Reporting Summary [file 41586_2023_6355_MOESM2_ESM.pdf]

nature portfolio

Corresponding author(s): Andrea Ganna
Last updated by author(s): Apr 20, 2023

Reporting Summary

Nature Portfolio wishes to improve the reproducibility of the work that we publish. This form provides structure for consistency and transparency in reporting. For further information on Nature Portfolio policies, see our Editorial Policies and the Editorial Policy Checklist.

Statistics

For all statistical analyses, confirm that the following items are present in the figure legend, table legend, main text, or Methods section.

n/a	Confirmed	
☐	☒	The exact sample size (*n*) for each experimental group/condition, given as a discrete number and unit of measurement
☐	☒	A statement on whether measurements were taken from distinct samples or whether the same sample was measured repeatedly
☐	☒	The statistical test(s) used AND whether they are one- or two-sided<br>*Only common tests should be described solely by name; describe more complex techniques in the Methods section.*
☐	☒	A description of all covariates tested
☐	☒	A description of any assumptions or corrections, such as tests of normality and adjustment for multiple comparisons
☐	☒	A full description of the statistical parameters including central tendency (e.g. means) or other basic estimates (e.g. regression coefficient) AND variation (e.g. standard deviation) or associated estimates of uncertainty (e.g. confidence intervals)
☐	☒	For null hypothesis testing, the test statistic (e.g. *F*, *t*, *r*) with confidence intervals, effect sizes, degrees of freedom and *P* value noted<br>*Give P values as exact values whenever suitable.*
☐	☒	For Bayesian analysis, information on the choice of priors and Markov chain Monte Carlo settings
☒	☐	For hierarchical and complex designs, identification of the appropriate level for tests and full reporting of outcomes
☐	☒	Estimates of effect sizes (e.g. Cohen's *d*, Pearson's *r*), indicating how they were calculated

Our web collection on statistics for biologists contains articles on many of the points above.

Software and code

Policy information about availability of computer code

Data collection	No code was used to collect data in the study.
Data analysis	Each individual study that contributed genetic-phenotype association summary statistics to the consortium carried out their association analyses independently of the consortium (study-specific information outlined in Supplementary Table 1). However, the consortium released phenotyping and analysis guidelines as a recommendation (https://www.covid19hg.org/). For quality control of genotype data we recommended using the Ricopili pipeline (PMID: 31393554). For genotype phasing and imputation we recommended the TopMed Imputation Server (PMID: 27571263) or Michigan Imputation Server (PMID: 27571263). For genome-wide association study (GWAS), we recommended SAIGE (PMID: 30104761), but some studies used PLINK (PMID: 17701901). Each study then submitted their GWAS summary statistics to the consortium for meta-analysis. No version was specified in the recommendation—each study used the best available version for them.<br>LD score regression v 1.0.1 [PMID: 25642630] was used for heritability and partitioned heritability analyses. Variants for Mendelian randomization instruments were selected using PLINK version 1.90b6.18 (PMID: 17701901). Exposure and outcome datasets were harmonized, and MR statistical analysis conducted using R version 4.0.3. with the R-package TwoSampleMR version 0.5.5 (PMID: 29846171) (which included a fixed-effect IVW analysis [PMID: 24114802], weighted median estimator [WME] [PMID: 27061298], weighted mode based estimator [WMBE] and MR Egger regression [PMID: 26050253]) and additionally MR-PRESSO version 1.0 (PMID: 29686387).<br>Code availability statement: The code for summary statistics lift-over, the projection PCA pipeline including precomputed loadings and meta-analyses are available at https://github.com/covid19-hg/; heritability estimation at https://github.com/AndrewsLabUCSF/COVID19_heritability; Mendelian randomization and genetic correlation at https://github.com/marcoralab/MRcovid; and subtype analyses at https://github.com/mjpirinen/covid19-hgi_subtypes.

For manuscripts utilizing custom algorithms or software that are central to the research but not yet described in published literature, software must be made available to editors and reviewers. We strongly encourage code deposition in a community repository (e.g. GitHub). See the Nature Portfolio guidelines for submitting code & software for further information.

Data

Policy information about availability of data

All manuscripts must include a data availability statement. This statement should provide the following information, where applicable:

- Accession codes, unique identifiers, or web links for publicly available datasets
- A description of any restrictions on data availability
- For clinical datasets or third party data, please ensure that the statement adheres to our policy

Summary statistics generated by COVID-19 HGI are available online, including per-ancestry summary statistics for African, Admixed American, East Asian, European, and South Asian ancestries (https://www.covid19hg.org/results/r7/). The analyses described here utilize the data release 7. If available, individual-level data can be requested directly from contributing studies, listed in Supplementary Table 1. We used publicly available data from GTEx v8 (https://gtexportal.org/home/; dbGaP Accession phs000424.v8.p2), the Neale lab (http://www.nealelab.is/uk-biobank/), Finucane lab (https://www.finucanelab.org), FinnGen Freeze 4 cohort (https://www.finngen.fi/en/access_results), and eQTL catalogue release 3 (http://www.ebi.ac.uk/eqtl/).

Human research participants

Policy information about studies involving human research participants and Sex and Gender in Research.

Reporting on sex and gender	Biological sex was collected by each study. The consortium recommended using biological sex as one of the covariates to correct for potential confounding effects (full recommended covariates are: age + age2 + sex + age*sex + 20 principal components obtained using genetic data). No sex or gender specific analysis was reported in this study. The consortium did not collect gender information.
Population characteristics	Summary statistics from 82 independent studies were included in consortium meta-analyses. The sample size for genetic ancestry populations for Sars-CoV2 infection was: n=17,898 Middle Eastern; n=53,116 South Asian; 40,580 East Asian; 148,891 African; 129,629 Ad-mixed American; 2,824,391 European.<br>Population characteristics regarding age, sex and exact case and control sample numbers for each contributing study are given in Supplementary Table 1.
Recruitment	The consortium pre-defined phenotype criteria for cases and controls, but the specific recruitment was carried out independently by each contributing study. COVID-19 disease status (critical illness, hospitalization status) was assessed following the Diagnosis and Treatment Protocol for Novel Coronavirus Pneumonia (PMID: 32358325). The critically ill COVID-19 group included patients who were hospitalized due to symptoms associated with laboratory-confirmed SARSCoV-2 infection and who required respiratory support or whose cause of death was associated with COVID-19. The hospitalized COVID-19 group included patients who were hospitalized due to symptoms associated with laboratory confirmed SARS-CoV-2 infection. The reported infection cases group included individuals with laboratory-confirmed SARSCoV-2 infection or electronic health record, ICD coding or clinically confirmed COVID-19, or self-reported COVID-19 (e.g. by questionnaire), with or without symptoms of any severity. Genetic ancestry-matched controls for the three case definitions were sourced from population-based cohorts, including individuals whose exposure status to SARS-CoV-2 was either unknown or infection-negative for questionnaire/electronic health record based cohorts.
Ethics oversight	Ethical statements for each contributing study are given in Supplementary Table 1.

Note that full information on the approval of the study protocol must also be provided in the manuscript.

Field-specific reporting

Please select the one below that is the best fit for your research. If you are not sure, read the appropriate sections before making your selection.

☒ Life sciences ☐ Behavioural & social sciences ☐ Ecological, evolutionary & environmental sciences

For a reference copy of the document with all sections, see nature.com/documents/nr-reporting-summary-flat.pdf

Life sciences study design

All studies must disclose on these points even when the disclosure is negative.

Sample size	The consortium meta-analysed genome-wide association study (GWAS) summary statistics from any individual study that had included a minimum of 100 cases and 100 controls in their analysis. The cutoff was aimed at reducing noise for the meta-analysis, but also to be inclusive of studies that had not yet accumulated large numbers of COVID-19 patient data. The consortium aimed to reach the largest possible sample size for the study and meta-analyzed all the submitted summary statistics. No statistical calculation for adequate sample size was performed, but the results identifying multiple genomic regions at genome-wide significance threshold indicates adequate power for genetic discovery.
Data exclusions	Individual level phenotype and genotype data exclusions were performed by each individual study, following the consortium analysis plan recommendations (www.covid19hg.org). Possible reasons for sample exclusion included removing genetic ancestry outliers within a study

	(using principal components analysis), poor quality of genetic data or lack of phenotypic data for a sample. The consortium manually examined GWAS summary statistics data submitted by each study (for each submitted analysis separately), including sample size used for analysis, allele frequency check against Gnomad reference panel, and distribution of test statistics. After meta-analysis, the results were checked for heterogeneity variant effects between contributing studies in Supplementary Table 2
Replication	No replication was performed. The consortium meta-analysed GWAS summary statistics, bringing together as many studies as possible to achieve the largest possible sample size and statistical power for association. this meant that the consortium included most large studies of COVID-19 host genetics that have been performed to date, so it was not possible to perform replication analyses in external cohorts. Therefore we performed manual checks on each study contributing summary statistics before entering them into the meta-analysis. In addition, after meta-analysis, we performed a check for heterogeneity between variant association estimates across studies contributing data. This allowed us to better understand whether the variant effects differed much between individual studies.
Randomization	No randomization was performed because there was no allocation of samples to experimental groups
Blinding	Blinding was not relevant to the study because no intervention was implemented in the study. The case status and severity of symptoms was evaluated for each sample by investigators from each study respectively. The consortium recommended using covariates to control for confounding: age + age2 + sex + age*sex + 20 principal components (obtained using genetic data) + study specific covariates (if any). The consortium meta-analysed summary statistics from these case/control studies, not individual level data. Details of which variables each study used and how the calculated PCs for their analysis are available in Supplementary Table 1.

Reporting for specific materials, systems and methods

We require information from authors about some types of materials, experimental systems and methods used in many studies. Here, indicate whether each material, system or method listed is relevant to your study. If you are not sure if a list item applies to your research, read the appropriate section before selecting a response.

Materials & experimental systems

n/a	Involved in the study
☒	☐ Antibodies
☒	☐ Eukaryotic cell lines
☒	☐ Palaeontology and archaeology
☒	☐ Animals and other organisms
☒	☐ Clinical data
☒	☐ Dual use research of concern

Methods

n/a	Involved in the study
☒	☐ ChIP-seq
☒	☐ Flow cytometry
☒	☐ MRI-based neuroimaging
